# Inhibition of WNT/β-catenin signalling during sex-specific gonadal differentiation is essential for normal human fetal testis development

**DOI:** 10.1186/s12964-024-01704-9

**Published:** 2024-06-15

**Authors:** Malene Lundgaard Riis, Gaspard Delpouve, John E. Nielsen, Cecilie Melau, Lea Langhoff Thuesen, Kristine Juul Hare, Eva Dreisler, Kasper Aaboe, Pia Tutein Brenøe, Jakob Albrethsen, Hanne Frederiksen, Anders Juul, Paolo Giacobini, Anne Jørgensen

**Affiliations:** 1 Department of Growth and Reproduction, Copenhagen University Hospital – Rigshospitalet, Copenhagen, Denmark; 2 International centre for Research and Research Training in Endocrine Disruption of Male Reproduction and Child Health (EDMaRC), Copenhagen University Hospital - Rigshospitalet, Copenhagen, Denmark; 3 Laboratory of Development and Plasticity of the Neuroendocrine Brain, Lille Neuroscience & Cognition, University of Lille, CHU Lille, UMR-S 1172, FHU 1000 days for health, Inserm, Lille, France; 4 Department of Obstetrics and Gynaecology, Hvidovre University Hospital, Hvidovre, Denmark; 5 Department of Gynaecology, Copenhagen University Hospital – Rigshospitalet, Copenhagen, Denmark; 6 Department of Obstetrics and Gynaecology, Copenhagen University Hospital - Herlev and Gentofte, Herlev, Denmark; 7 Department of Clinical Medicine, University of Copenhagen, Copenhagen, Denmark; 8 Division of Translational Endocrinology, Department of Endocrinology and Internal Medicine, Copenhagen University Hospital - Herlev and Gentofte, Herlev, Denmark

**Keywords:** Human fetal gonads, Sex-specific development, WNT/β-catenin signalling, Ex vivo culture, Ovarian and testicular differentiation, Germ cell development, Supporting cell lineages

## Abstract

Sex-specific gonadal differentiation is directed by complex signalling promoting development in either male or female direction, while simultaneously inhibiting the opposite pathway. In mice, the WNT/β-catenin pathway promotes ovarian development and the importance of actively inhibiting this pathway to ensure normal testis development has been recognised. However, the implications of alterations in the tightly regulated WNT/β-catenin signalling during human fetal gonad development has not yet been examined in detail. Thus, the aim of this study was to examine the consequences of dysregulating the WNT/β-catenin signalling pathway in the supporting cell lineage during sex-specific human fetal gonad development using an established and extensively validated ex vivo culture model. Inhibition of WNT/β-catenin signalling in human fetal ovary cultures resulted in only minor effects, including reduced secretion of RSPO1 and reduced cell proliferation although this was not consistently found in all treatment groups. In contrast, promotion of WNT/β-catenin signalling in testes severely affected development and function. This included disrupted seminiferous cord structures, reduced cell proliferation, reduced expression of SOX9/AMH, reduced secretion of Inhibin B and AMH as well as loss of the germ cell population. Additionally, Leydig cell function was markedly impaired with reduced secretion of testosterone, androstenedione and INSL3. Together, this study suggests that dysregulated WNT/β-catenin signalling during human fetal gonad development severely impairs testicular development and function. Importantly, our study highlights the notion that sufficient inhibition of the opposite pathway during sex-specific gonadal differentiation is essential to ensure normal development and function also applies to human fetal gonads.

**Supplementary Information:**

The online version contains supplementary material available at 10.1186/s12964-024-01704-9.

## Introduction

In early embryonic development, the bipotential gonads emerge from the intermediate mesoderm [1] and differentiate into either ovaries or testes directed by cues from sex-specific signalling pathways (Reviewed in [2–5]). The bipotential gonads initially contain somatic precursor cells, which includes the supporting cells that develop into granulosa cells in ovaries and Sertoli cells in testes as well as the steroidogenic cells that develop into theca cells in ovaries and Leydig cells in testes. Subsequently, the primordial germ cells (PGCs) which are specified from the proximal epiblast migrate via the hindgut to arrive in the developing bipotential gonads [6]. At this early developmental stage, the PGCs are bipotential and retain the potential to differentiate into oogonia or gonocytes depending on the signals received from the surrounding somatic niche (Reviewed in [2–5]).

In XY gonads, the Y-chromosome encoded gene *SRY* induce upregulation of SOX9 [7, 8], which together with FGF9 drives the sex-specific differentiation of the supporting cells into Sertoli cells [9–11]. Subsequently, the Sertoli cells promote differentiation of the steroidogenic Leydig cells and together they form the somatic niche that supports the germ cell population and direct germ cell commitment to the male developmental pathway (Reviewed in [4, 5, 12, 13]). While it is well established that *SRY* is the main determinant of testicular development, the initiation of ovarian development is less well understood, although a subset of genes has been identified in recent years [14–20], including the factor WT1-KTS, which was recently identified to be a key determinant of female sex determination [21]. Studies in mice have also demonstrated that the early pro-ovarian factors RSPO1, WNT4 and downstream β-catenin signalling promote differentiation of supporting precursor cells into (pre)granulosa cells [14–20], thereby directing the development of the bipotential gonads towards ovarian fate. Importantly, the signalling pathways promoting testicular development (SOX9/FGF9) and ovarian development (WNT/β-catenin) act antagonistically on the opposing pathway to maintain the initially established fate of the supporting cell lineage [2, 3]. Hence, in the absence of a WNT signal in XY gonads, β-catenin is targeted for proteasomal degradation ensuring termination of WNT signalling [22], whereas stabilization of β-catenin and thus active WNT signalling in supporting cells in XY gonads is sufficient to promote male-to-female sex reversal in mice [14].

The sex-specific fate decision in the supporting cells of developing gonads results in a cascade of events including morphological changes and differential production of hormones and autocrine/paracrine factors, which ultimately lead to the development of either testes or ovaries (Reviewed in detail in [4, 5, 23]). Consequently, alterations in the development, differentiation, or maintenance of the supporting cells lineage and the function of the somatic cells can lead to differences of sex development (DSD), a heterogenous group of congenital variations in the genetic, gonadal, or anatomical sex. Interestingly, loss-of-function variants in *RSPO1* or *WNT4* in 46,XX individuals has been associated with female-to-male reversal or in the latter case in virilisation and Müllerian-duct regression [24–26]. Based on information from these patients, it has been suggested that WNT/β-catenin signalling may also be important in the promotion of ovarian development in humans. Accordingly, mutations in *ZNRF3* that normally antagonizes WNT signalling has been reported to result in male-to-female reversal in 46,XY individuals [27], indicating that repression of WNT/β-catenin may be essential for human testicular development. Nonetheless, the exact role of WNT/β-catenin signalling in sex-specific human fetal gonad differentiation remains largely unexplored.

Therefore, this study aimed to investigate the consequences of manipulating WNT/β-catenin signalling in the supporting cell lineages during early human fetal gonad development using an established and extensively validated ex vivo  culture model [28–30]. The present study demonstrates that dysregulation of the WNT/β-catenin signalling pathway during early human fetal gonad development impairs the sex-specific differentiation of the somatic niche with most pronounced effects on testicular development and function, including alterations in both the supporting and steroidogenic cell lineages as well as effects subsequently extending to the germ cell population.

## Methods

### Collection of human fetal gonads and ethical approval

Human fetal gonads were collected from elective terminations of first trimester pregnancies. The elective terminations were carried out at the Departments of Gynaecology at Copenhagen University Hospital (Rigshospitalet), Hvidovre Hospital and Herlev Hospital, Denmark. The study was approved by the regional ethics committee (# H-1-2012-007). All women gave their consent after being given oral and written information about the project. None of the terminations were for pathological reasons of the pregnancy or fetal abnormality. A total of 117 gonads was included in this study with age ranging from 6 to 10 post conceptional weeks (PCW) from 41 female foetuses and 30 male foetuses. Fetal age was calculated by scanning crown-rump length and by foot-length evaluation [31]. The nomenclature used to describe fetal age of samples was PCW followed by days (e.g., 8 + 5 PCW) and indicate the age at the time of ex vivo culture setup. The sex of the foetus was determined by quantitative RT-PCR using QuantStudio3 (Applied biosystems, Thermo Fischer Scientific, Denmark). DNA was isolated from surplus fetal tissue using NucleoSpin Tissue kit (MACHEREY NAGEL, 740952.250) as described by the manufacturer. Quantitative RT-PCR analysis of *SRY* gene expression (forward sequence: GAATATTCCCGCTCTCCGGA, reverse sequence: GCTGGTGCTCCATTCTTGAG) was conducted in duplicates with SYBR Green master mix (Agilent, 600828-51) and *ZFYX* gene expression (forward sequence: ACCRCTGTACTGACTGTGATTACAC, reverse sequence: GCACYTCTTTGGTATCYGAGAAAGT) was included as positive control. Additionally, control samples were included for both XY tissue and XX tissue in each analysis.

### Ex vivo gonad tissue culture

Human fetal gonads were cultured using the ex vivo culture model as previously described [28–30]. In brief, human fetal gonads were dissected in ice-cold phosphate buffered saline (PBS) and cut into 1 mm^3^ tissue fragments. The tissue fragments were subsequently cultured for 24 h awaiting sex determination in 37°C and 5% CO_2_ in a hanging drop of 40 µl media consisting of MEMα medium (Gibco) supplemented with 1 × MEM non-essential amino acids, 2 mM sodium pyruvate, 2 mM L-Glutamine, 1 × Insulin-Transferrin-Selenium (ITS) supplement (Sigma-Aldrich), 1 × penicillium/streptomycin and 10% Fetal bovine serum. All supplements were from Gibco (Nærum, Denmark), except ITS (Sigma-Aldrich, Brøndby, Denmark).

To manipulate with WNT/β-catenin signalling in human fetal gonads, two small molecule inhibitors were used; IWR-1 (which stabilizes the β-catenin destruction complex and thus ensures degradation of β-catenin) in fetal ovaries and CHIR99021 (which inhibits GSK-3 that normally ensures degradation of β-catenin in the absence of a WNT signal) in fetal testes. Following sex determination of the foetus, gonadal tissue fragments from female foetuses were thus transferred to a new hanging drop of media supplemented with either: (1) 1 µM IWR-1, abbreviated IWR (2) 1 µM IWR-1 + 50 ng/ml FGF9, abbreviated IWR+FGF9 or (3) 1 µM IWR-1 + 50 ng/ml FGF9 + 25 ng/ml Activin A + 25 ng/ml Activin B + 25 ng/ml TGFβ1, abbreviated IWR+FAT (See Table 1 for details). Gonadal tissue fragments from male foetuses were transferred to media supplemented with either: (1) 3 µM CHIR99021, abbreviated CHIR (2) 3 µM CHIR99021 + 100 ng/ml RSPO1, abbreviated CHIR+RSPO1 or (3) 3 µM CHIR99021 + 100 ng/ml RSPO1 + 100 ng/ml WNT4, abbreviated CHIR+RW. For all treatment setup at least one tissue fragment from each foetus was cultured as vehicle control (either with 0.1% DMSO, 0.1% BSA in PBS or 0.1% DMSO, 0.1% BSA, 4mM HCl in PBS). Importantly, due to a considerable biological variation between the biological replicates (foetuses), mainly due to differences in the time from the surgical termination of pregnancy until the tissue fragments can be setup in ex vivo cultures, all treatment-induced changes are shown as a ratio relative to vehicle control treated samples from the same foetus. The doses selected and used were based similar types of ex vivo culture studies in mice [19, 32] and on pilot experiments in which human fetal testis (*n* = 3) and ovaries (*n* = 3) were cultured ex vivo with the treatment doses and combinations of pharmaceutical inhibitors and recombinant proteins listed above. Media from these pilot experiments were initially analysed for effects on the secretion of AMH and Inhibin B (tendency to treatment-induced increase in fetal ovaries and decrease in fetal testes). Gonadal tissue was cultured for 14 days with a complete media change every second day. At the end of ex vivo culture, tissue fragments were either fixed in formalin and subsequently paraffin embedded for immunohistochemistry/immunofluorescence analysis or fixed in 4% PFA for 3D imaging analysis. The media were collected and pooled for each individual tissue fragment throughout the culture period and was stored at -20 °C until further analysis.

**Table 1:** Media supplements

Small-molecule inhibitors	Concentration used	Manufacturer	Product number	Note
IWR-1	1 µM	Sigma-Aldrich	I0161-5MG	
CHIR99021	3 µM	Tocris	4423	
**Recombinant proteins**				
FGF9	50 ng/ml	Sigma-Aldrich	SRP3040	
Activin A	25 ng/ml	R&D Systems	338-AC-010	
Activin B	25 ng/ml	R&D Systems	659-AB-005	
TGFβ1	25 ng/ml	R&D Systems	240-B-002	
RSPO1	100 ng/ml	R&D Systems	4645-RS-025	
WNT4	100 ng/ml	R&D Systems	6076-WN-005	Product discontinued

### Immunohistochemistry

Immunohistochemical analysis of gonadal tissue was conducted as previously described [33], with few modifications. In brief, paraffin sections (4 μm) of formalin fixed tissue were deparaffinized and rehydrated. Antigen retrieval was accomplished in a pressure cooker (medical decloaking chamber, Biocare, Concord, CA, USA) at 110°C for 30 min in retrieval buffer or by microwaving sections for 15 min in retrieval buffer. Blocking of endogenous peroxidase was performed with 1% (v/v) H_2_O_2_ in methanol for 30 min. Sections were incubated with 0.5% (w/v) milk powder in Tris buffered saline (TBS). After incubation overnight at 4°C with primary antibody (details listed in Supplementary Table 1), the sections were incubated with the appropriate secondary antibody (ImmPRESS, MP-7401, MP-7402, MP-7405, Vector Laboratories, CA, USA) for half an hour at RT and signal development was performed with AEC (ImmPACT, SK-4205, Vector Laboratories, CA, USA) visualised as red staining. The sections were washed with TBS between the incubation steps except between blockade for cross-reactivity and the primary antibodies. In all experiments positive controls were included as well as negative controls in which the primary antibody was replaced by the dilution buffer alone. None of the negative control sections showed any staining. Counterstaining was conducted with Mayer’s haematoxylin.

### Immunofluorescence

Immunofluorescence was performed on ex vivo cultured tissue as previously described [28]. In brief, immunofluorescence was performed with TBS washes (3 × 5 min) between each step and all incubations were carried out in in a humidity box (Fisher Scientific, UK). Sections (4 μm) of formalin fixed tissue were dewaxed and rehydrated using standard procedures, followed by heat-induced antigen retrieval (pressure cooker) in 0.01 M citrate buffer (pH 6) and peroxidase block in 3% (v/v) H_2_O_2_ in methanol for 30 min. Next, the sections were blocked in normal chicken serum (NCS; Biosera, Ringmer, UK) diluted 1:5 in TBS containing 5% (w/v) BSA (NCS/TBS/BSA), followed by incubation with COUPTF-II antibody diluted in NCS/TBS/BSA overnight at 4°C. The next day, sections were incubated with peroxidase-conjugated chicken anti-mouse secondary antibody (Santa Cruz), diluted 1:200 in NCS/TBS/BSA for 30 min at room temperature (RT), and followed by incubation with Tyr-Cy3 (Perkin Elmer-TSAPlus Cyanine3 System; Perkin Elmer Life Sciences, Boston, MA, USA) according to manufacturer’s instructions. Before the next primary antibody dilution was added, the sections were again subjected to antigen retrieval by blocking in NCS/TBS/BSA and overnight incubation at 4°C with AMH or OCT4 antibody diluted in NCS/TBS/BSA. On the third day, slides were incubated with peroxidase-conjugated appropriate secondary antibody (Santa Cruz) diluted 1:200 in NCS/TBS/BSA for 30 min at RT, followed by incubation with Tyr-Fluorescein (Perkin Elmer-TSA-Plus Fluorescein System; Perkin Elmer Life Sciences) according to manufacturer’s instructions. Sections were again subjected to antigen retrieval followed by blocking in NCS/TBS/BSA and incubation with SOX9 or FOXL2 antibodies diluted in NCS/TBS/BSA overnight at 4°C. Sections were then incubated with peroxidase-conjugated chicken anti-rabbit secondary antibody (Santa Cruz), diluted 1:200 in NCS/TBS/BSA for 30 min at RT, followed by incubation with Tyr-Cy5 (Perkin Elmer-TSA-Plus Cyanine5 System; Perkin Elmer Life Sciences, Boston, MA, USA) according to manufacturer’s instructions. Sections were counterstained with DAPI (Sigma-Aldrich) diluted 1:500 in TBS for 10 min. Finally, slides were mounted with Permafluor (Thermo Scientific, UK) and fluorescent images captured using an Olympus BX61 microscope (Olympus). Antibody dilutions are listed in Supplementary Table 2.

#### BrdU incorporation

Before end of ex vivo culture period, tissue fragments were cultured with BrdU labeling agent (Life Technologies, Nærum, Denmark) diluted 1:10 in media for 6 h to allow for the detection of proliferating cells in the tissue. After 6 h, tissue fragments were formalin fixed and paraffin embedded as described above. Proliferating cells were visualized by immunohistochemical analysis using a BrdU antibody (Supplementary Table 1) as described in the Immunohistochemistry section.

### Quantification of cells

The number of OCT4^+^ (gonocyte and oogonia marker), BrdU^+^ (proliferation marker) and cPARP^+^ (apoptosis marker) cells were quantified per area of tissue using one entire tissue section. The area of fetal gonadal tissue was calculated following scanning of sections on a NanoZoomer 2.0 HT (Hamamatsu Photonics, Germany) and images captured using the software NDPview version 2.6.13 (Hamamatsu Photonics). Images from the NanoZoomer were subsequently analysed using the counting tool in Adobe Photoshop CC version 20.0.1. For all quantifications, tissue samples from at least five embryos/foetuses were included.

### 3D imaging and quantitative analysis

For 3D imaging analysis, ex vivo cultured tissue was washed in ice-cold PBS and fixed in 4% PFA for 2–4 h before it was transferred to PBS containing 0.01% Sodium Azide (Sigma-Aldrich) and kept at 4°C until iDISCO + whole-mount immunostaining analysis which was carried out as previously described [34] and detailed below.

### Sample pre-treatment with methanol

As previously described [35, 36] samples were embedded in 2% agarose (Roth) prepared in 1× PBS (Invitrogen) prior to clearing and processing. Samples were then dehydrated for 1 h at RT in ascending concentrations of methanol in water (20%, 40%, 60%, 80% and 100% two times). Next, samples were incubated in dichloromethane 66% (Sigma-Aldrich #270,997) with 33% methanol overnight at 4°C. After 2 washes of 1 h with 100% methanol, samples were incubated at 4°C with the bleaching solution (5% H_2_O_2_ in methanol) to remove the pigmentation. Samples were then rehydrated in descending concentrations of methanol (100% × 2, 90%, 80%, 70%, 60% and 50%) and stored in 1× PBS at 4°C.

### Whole-mount immunostaining

Samples were incubated at room temperature (RT) on an adjustable rotator in a permeabilized blocking solution (PBSGT) of 1 × PBS containing 0.2% gelatin (Sigma), 1% Triton X-100 (Sigma-Aldrich) and 0.01% Sodium Azide for two nights. For immunostaining, samples were transferred in PBSGT together with the primary antibodies SOX9 (rabbit polyclonal antibody), AMH (mouse monoclonal antibody), and Cytochrome P450 17A1/CYP17A1 (goat polyclonal antibody) and incubated at 37°C in agitation for one week. This was followed by 6 washes of 1 h in PBSGT at RT. Samples were then incubated in secondary antibodies (anti-rabbit AlexaFluor 488, anti-mouse AlexaFluor 568 and anti-Goat AlexaFluor 647, Thermofisher) diluted at 1:500 in 1×PBS containing 0.2% gelatin (Prolabo), and 0.5% Triton X-100 (Sigma-Aldrich) (PBSGT) at RT for three days. After six washes of 30 min in PBSGT at RT, samples were stored in the dark at 4°C until tissue clearing. Antibody details and dilutions are listed in Supplementary Table 3.

### Tissue clearing

All incubation steps were performed at RT in a fume hood, on a tube rotator at 14 rpm covered with aluminium foil to avoid contact with light. Samples were dehydrated in a graded series (20%, 40%, 60%, 80% and 100% × 2) of methanol diluted in PBS × 1. Next, samples were incubated in dichloromethane 66% with 33% ethanol overnight at 4 °C. This was followed by a delipidation step of 45 min in 100% dichloromethane (DCM; Sigma-Aldrich). Samples were cleared in dibenzyl ether (DBE; Sigma-Aldrich) for 2 h at RT on constant agitation and in the dark. The next day, samples were stored in individual light-absorbing glass vials (Rotilabo, Roth) at RT. In these conditions, samples could be stored and imaged for up to 9 months without any significant fluorescence loss.

### Imaging

3D imaging was performed as previously described [37]. An ultramicroscope (LaVision BioTec) and an Andor Neo 5.5 sCMOS camera using InspectorPro software (LaVision BioTec) were used to perform imaging. The light sheet was generated by a laser (wavelength 568–647 nm, Coherent Sapphire Laser, LaVision BioTec) and two cylindrical lenses with a 1.1×/0.1NA and 4×/0.3NA objectives were used. Samples were placed in an imaging reservoir made of 100% quartz (LaVision BioTec) filled with DBE and illuminated from the side by the laser light. The step size between each image was fixed at 4 μm. Analysis of 3D imaging was performed using the Imaris software (version 9.9, Bitplane, Oxford Instruments).

### 3D analysis

Images, 3D volume, and movies were generated using Imaris ×64 software (version 9.9.0, Bitplane). Stack images were first converted to imaris file (.ims) using ImarisFileConverter and 3D reconstruction was performed using “volume rendering”. Optical slices of samples were obtained using the “orthoslicer” tools. The “surface” tool was used and precisely the machine learning plugin Labkit (pixel classification) from Fiji (National Institute of Health, Bethesda) to segment SOX9 positive cells and remove the background noise. After several machine-learning based training sessions for pixel classification, the classifier of segmentation was applied to all 3D acquisitions. This was followed by the use of the « spots » tool to automatically count the SOX9 positive cells of the new channel after image segmentation. The number of cells was obtained by the tab « statistics ». The staining of AMH was segmented using Labkit Fiji plugin. In this case, the quantification was done on the volume of AMH inside the mask previously created. This information was extracted by the tab « statistics » of « surface » tool in µm^3^.

### RSPO1 ELISA assay

RSPO1 measurements were performed on media from ex vivo tissue cultures using Human Rspondin-1 ELISA Kit (R&D Systems, DY4546-05) according to the manufacturers instructions. RRID: AB_2936293. Media samples were diluted 25% (75 µl media sample + 25 µl culture media). The detection limit of RSPO1 assay was 31.1 pg/ml and the intra-assay variation was ≤ 10% for all controls except one control in each of the two batches, for those the intra-assay variance was below ≤ 15%.

### AMH hormone measurements

Quantification of AMH concentration was measured in ex vivo culture media by ELISA using the Beckman Coulter ACCESS AMH assay Reagents Kit (Ref. B13127) and Calibrator kit (Ref. B13128) as previously described [28]. RRID: AB_2892998. Collected media samples were diluted 1:10 in culture media prior to analysis, with additional sample dilution (1:25) and (1:50) necessary for a few of the samples. The detection limit of the AMH assay was 0.14 pmol/L and the intra-assay variation was ≤ 9%.

### Inhibin B hormone measurements

Inhibin B concentration in ex vivo culture media was measured by ELISA using the Beckman Coulter INHIBIN B GEN II ELISA assay Reagents Kit (Ref. A81303) and Calibrator kit (Ref. A81304) as previously described [28]. RRID: AB_2827405. Collected media samples were diluted 1:50 for samples originating from male ex vivo tissue cultures and 1:20 for samples from female ex vivo tissue cultures. The detection limit of the inhibin B assay was 3 pg/ml, and the intra-assay variation was ≤ 10%.

### Androgen measurements

Quantification of androgens; testosterone, androstenedione, and dehydroepiandrosterone sulphate (DHEAS) in media from ex vivo cultures were performed using a highly sensitive LC-MS/MS method as previously described [38], and adjusted for measurement in culture media [28]. In brief, the modifications were the following: calibration curves were prepared in culture media, control samples were prepared by spiking with high and low concentration of steroids, and all collected media samples were diluted (1:4) in culture media prior to analysis. Each batch included standards for calibration curves, approximately 20 unknown samples, one blank and three pooled controls spiked with steroid standards at low and high levels. Limit of quantification was 0.012 nM for testosterone, 0.042 nM for androstenedione and 19 nM for DHEAS. The inter-day variation expressed as the relative standard deviation (RSD) was ≤ 14% for controls spiked in low levels and ≤ 3% for controls spiked in high levels.

### INSL3 measurements by LC-MS/MS

Quantification of INSL3 in media from ex vivo cultures was performed using LC-MS/MS as previously described [39], with the exception that INSL3 calibrators were diluted in culture media instead of serum. Limit of detection was 0.03 µg/l and limit of quantification was 0.15 µg/l. Intra-assay variation was ≤ 10%.

### Statistics

Due to the substantial heterogeneity between the biological replicates (foetuses), a paired analysis approach was used in which treated samples were always compared to vehicle control treated samples from the same foetus. For vehicle controls, the mean of all tissue fragments from each fetus was set to 1 and the individual ratio for each was calculated. Statistical analysis was performed using the ratio paired t-test in GraphPad Prism software version 8, which utilizes the logarithm of the ratio to test the difference between a given control and treatment group. The ratio paired t-test was performed individually for each of the treatments compared with vehicle controls. This was due to the small amount of tissue available for the youngest foetuses which only allowed the setup of one treatment group. The data are shown as ratios compared with the internal controls and illustrated for each treatment group as individual data points and mean ± SEM, with the indicated “n” corresponding to the number of foetuses. Asterisk indicates statistical significance with * *P* < 0.05, ** *P* < 0.01, *** *P* < 0.001 and **** *P* < 0.0001.

## Results

### Stimulation of WNT/β-catenin signalling in ex vivo cultures of human fetal testes does not affect apoptosis but results in reduced proliferation

**Fig. 1:**
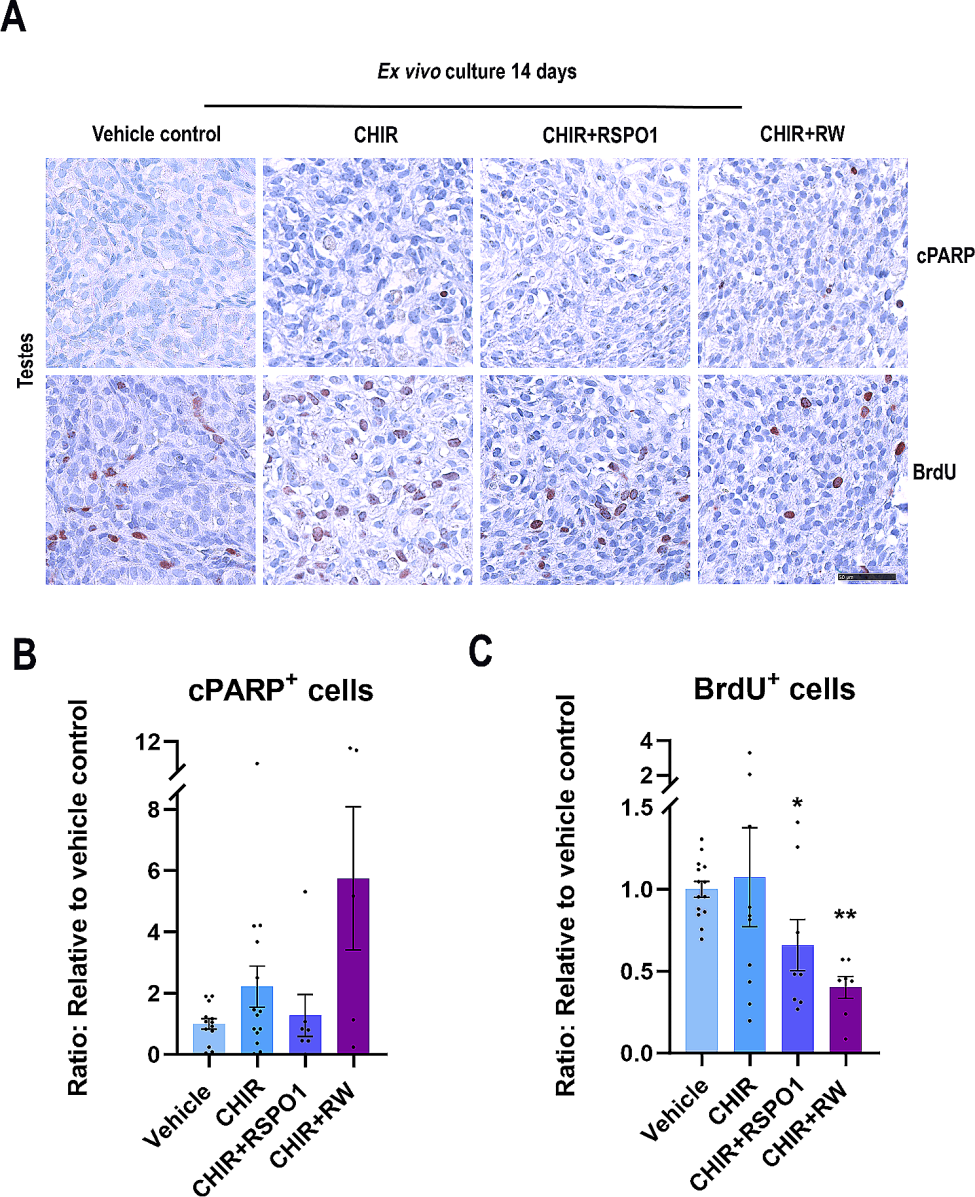
Stimulation of WNT/β-catenin signalling in ex vivo cultures of human fetal testes did not affect apoptosis but resulted in reduced proliferation. **(A)** Representative images of cPARP (apoptosis marker) and BrdU (proliferation marker) immunostaining in ex vivo cultured fetal testes treated with CHIR (3 µM), CHIR + RSPO1: CHIR (3 µM) + RSPO1 (100 ng/ml) or CHIR + RW: CHIR (3 µM) + RSPO1 (100 ng/ml) + WNT4 (100 ng/ml). Counterstaining was performed with Mayer’s haematoxylin; scale bar 50 μm. Age of fetal samples shown (at start of experiment): Vehicle control 8 + 2 PCW; CHIR 9 + 2 PCW; CHIR + RSPO1 8 + 1 PCW; CHIR + RW 7 + 4 PCW. **(B)** Quantification of cPARP-positive cells/mm^2^ in ex vivo cultured fetal testes treated with CHIR (*n* = 14), CHIR + RSPO1 (*n* = 7) or CHIR + RW (*n* = 5). **(C)** Quantification of BrdU-positive cells/mm^2^ in ex vivo cultured fetal testes treated with CHIR (*n* = 10), CHIR + RSPO1 (*n* = 8) or CHIR + RW (*n* = 7). Results are shown as fold change compared to internal vehicle control with data presented as mean ± SEM with individual datapoints included. Asterisk indicates statistical significance compared to vehicle control * *P* < 0.05 and ** *P* < 0.01

Promotion of WNT/β-catenin signalling in human fetal testes was achieved following treatment with CHIR99021 (GSK-3 inhibitor which in the absence of a WNT signal degrades β-catenin). To further promote stimulation of WNT/β-catenin signalling, treatment with CHIR was combined with recombinant proteins for granulosa cell factors, either RSPO1 alone or RSPO1 + WNT4 (RW). Initially, the effects on proliferation and apoptosis were examined in the ex vivo cultured testes. Overall, few apoptotic (cPARP^+^) cells were detected regardless of treatment (Fig. 1A), and proliferating (BrdU^+^) cells were observed in all treatment groups during culture. Together with the overall good preservation of tissue morphology, this suggests that the treatments and doses were not inducing cytotoxic effects (Fig. 1A). Quantification of cPARP^+^ cells/mm^2^ (Fig. 1B) and BrdU^+^ cells/mm^2^ (Fig. 1C) demonstrated that treatment did not affect apoptosis, while proliferation was reduced in fetal testes treated with CHIR + RSPO1 (34%, *p* < 0.05) and CHIR + RW (60%, *p* < 0.01). A tendency towards a higher level of apoptosis was also observed in testes treated with CHIR + RW, although this was not statistically significant. Together, these results suggest that promotion of WNT/β-catenin signalling in ex vivo cultured fetal testes negatively affects proliferation but not apoptosis.

### Inhibition of WNT/β-catenin signalling in ex vivo cultures of human fetal ovaries does not affect apoptosis but results in reduced proliferation

**Fig. 2:**
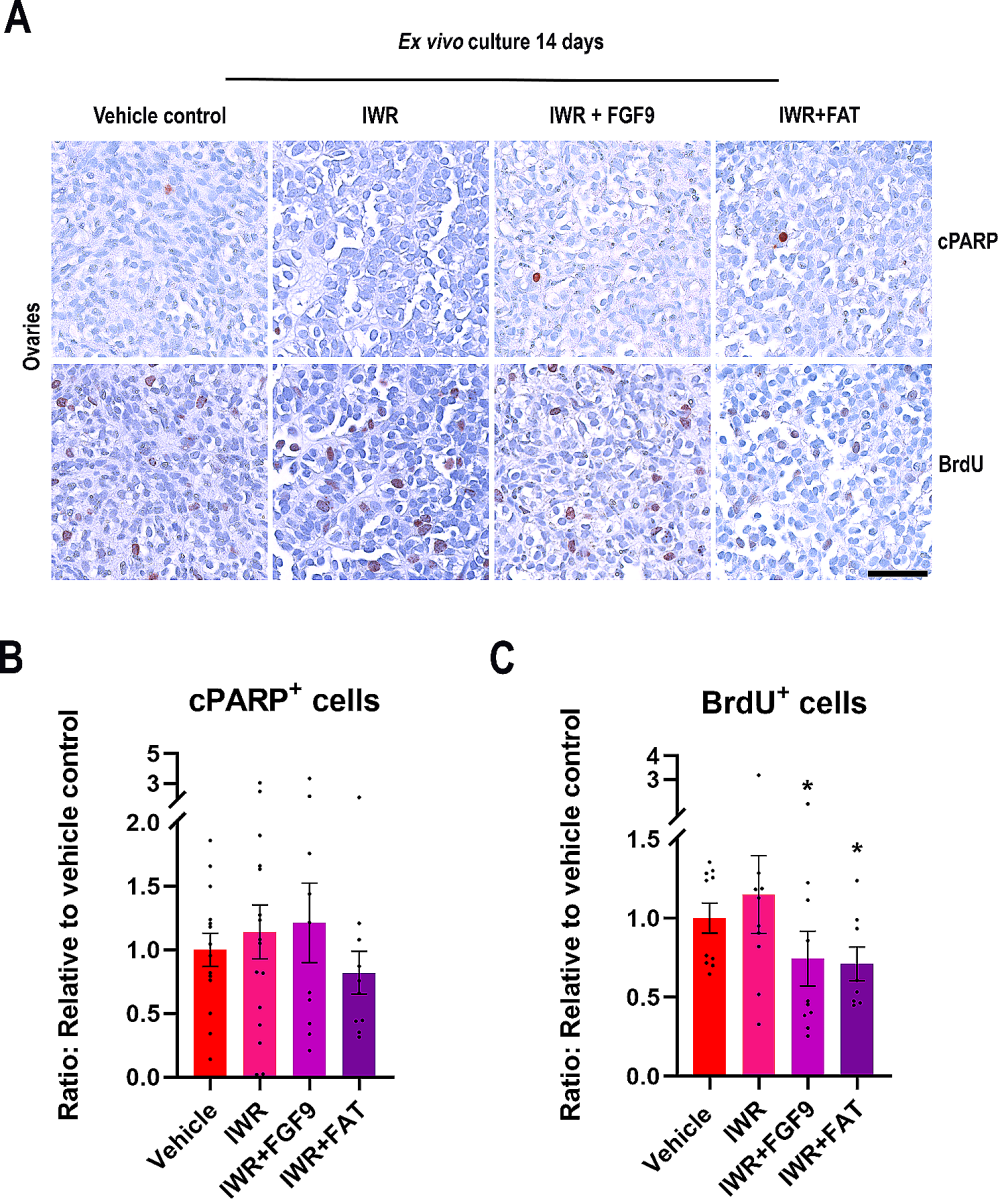
Inhibition of WNT/β-catenin signalling in ex vivo cultures of human fetal ovaries did not affect apoptosis but resulted in reduced proliferation. **(A)** Representative images of cPARP (apoptosis marker) and BrdU (proliferation marker) immunostaining in ex vivo cultured fetal ovaries treated with IWR (1µM), IWR + FGF9: IWR-1 (1µM) + FGF9 (50 ng/ml) or IWR + FAT: IWR-1 (1µM) + FGF9 (50 ng/ml) + Activin A (25 ng/ml) + Activin B (25 ng/ml) + TGFβ (25 ng/ml). Counterstaining was performed with Mayer’s haematoxylin; scale bar 50 μm. Age of fetal samples shown (at start of experiment): Vehicle control 8 + 2 PCW; IWR 9 + 3 PCW; IWR + FGF9 8 + 2 PCW; IWR + FAT 8 + 0 PCW. **(B)** Quantification of cPARP-positive cells/mm^2^in ex vivo cultured fetal ovaries treated with vehicle control, IWR (*n* = 16), IWR + FGF9 (*n* = 10) or IWR + FAT (*n* = 10). **(C)** Quantification of BrdU-positive cells/mm^2^ in ex vivo cultured fetal ovaries treated with vehicle control, IWR (*n* = 10), IWR + FGF9 (*n* = 10) or IWR + FAT (*n* = 8). Results are shown as fold change compared to internal vehicle control with data presented as mean ± SEM with individual datapoints included. Asterisk indicates statistical significance compared to vehicle control * *P* < 0.05

Inhibition of WNT/β-catenin signalling in fetal ovaries was performed by treatment with IWR-1, which stabilise the β-catenin destruction complex. IWR-1 treatment was combined with recombinant proteins for Sertoli cell factors, either FGF9 alone or a combination of FGF9 + Activin A + Activin B + TGFβ (FAT). In general, few apoptotic (cPARP^+^) cells were observed in the ex vivo cultured fetal ovaries irrespectively of treatment (Fig. 2A). Similar to the observations in ex vivo cultured testes, numerous proliferative (BrdU^+^) cells were detected in ex vivo cultured human fetal ovaries in all treatment groups (Fig. 2A), thereby suggesting that the treatments and selected doses did not induce cytotoxic effects. Quantification of the number of cPARP^+^ cells/mm^2^ (Fig. 2B) showed no effect of either of the treatments. In contrast, proliferation (BrdU^+^ cells/mm^2^) was reduced following treatment with IWR + FGF9 (26%, *p* < 0.05) and IWR + FAT (29%, *p* < 0.05) (Fig. 2C). Collectively, these results indicate that inhibition of WNT/β-catenin in ex vivo cultured fetal ovaries combined with exposure to recombinant Sertoli cell factors does not influence apoptosis but reduce cell proliferation in the fetal ovary cultures.

### Stimulation of WNT/β-catenin signalling in ex vivo cultures of human fetal testes affect sertoli cell identity and function

**Fig. 3:**
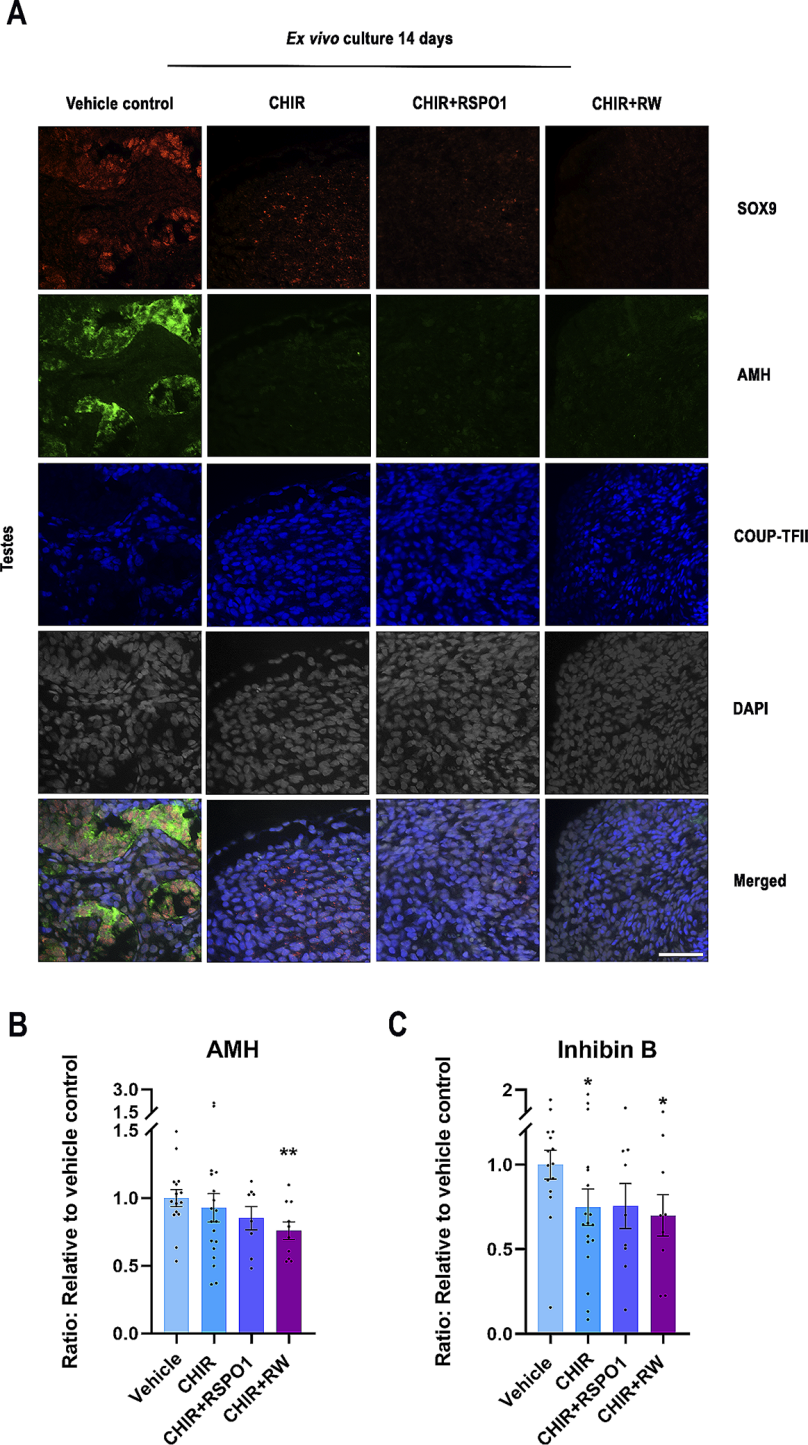
Stimulation of WNT/β-catenin signalling in ex vivo cultures of human fetal testes affected Sertoli cell identity and function. **(A)** Representative images of triple immunofluorescence staining of SOX9 (Sertoli cell marker, red), AMH (Sertoli cell marker, green), COUPTF-II (Interstitial cell marker, blue) and DAPI (grey) in ex vivo cultured fetal testes treated with CHIR (3 µM), CHIR + RSPO1: CHIR (3 µM) + RSPO1 (100 ng/ml) or CHIR + RW: CHIR (3 µM) + RSPO1 (100 ng/ml) + WNT4 (100 ng/ml). Age of fetal samples shown (at start of experiment): Vehicle control 9 + 0 PCW; CHIR 9 + 0 PCW; CHIR + RSPO1 9 + 4 PCW; CHIR + RW 9 + 0 PCW. Scale bar corresponds to 50 μm. **(B)** Secretion of AMH measured in media from ex vivo cultured fetal testes treated with CHIR (*n* = 19), CHIR + RSPO1 (*n* = 9) or CHIR + RW (*n* = 10). **(C)** Inhibin B measured in media from ex vivo cultured fetal testes treated with CHIR (*n* = 18), CHIR + RSPO1 (*n* = 9) or CHIR + RW (*n* = 9). Results are shown as fold change compared to internal vehicle control with data presented as mean ± SEM with individual datapoints included. Asterisk indicates statistical significance compared to vehicle control with * *P* < 0.05 and ** *P* < 0.01

The effects on Sertoli cells were examined after promoting WNT/β-catenin signalling in the fetal testis. The expression of the Sertoli cell markers SOX9 (nuclear) and AMH (cytoplasmic) was examined together with the interstitial cell marker COUP-TFII (nuclear) (Fig. 3A). Treatment with CHIR, CHIR + RSPO and CHIR + RW resulted in reduced expression of both SOX9 and AMH in ex vivo cultured fetal testes with the most pronounced effects observed in testis cultures treated with CHIR + RW. Notably, no expression of the granulosa cell marker FOXL2 was induced in the ex vivo cultured testes following treatment (Supplementary Fig. 1). Importantly, the seminiferous cord structure appeared to be impaired following treatment with CHIR, CHIR + RSPO1 and CHIR + RW, with no or only few seminiferous cord structures observed (Supplementary Fig. 2).

The stimulation of WNT/β-catenin signalling in ex vivo cultured fetal testes did not result in the production of detectable levels of the granulosa cell factor RSPO1 in any of the treatment groups. However, treatment with CHIR + RW reduced the secretion of AMH (24%, *p* < 0.05) compared with vehicle controls and likewise the secretion of Inhibin B was reduced after treatment with CHIR (26%, *p* < 0.05) and CHIR + RW (29%, *p* < 0.05) (Fig. 3B). Treatment with CHIR + RSPO1 also appeared to reduce the secretion of both AMH and Inhibin B (Fig. 3B), but the effect was not statistically significant. Together these results suggest that stimulation of WNT/β-catenin signalling in ex vivo cultured fetal testes affects Sertoli cell development and function as observed by a reduced expression of SOX9 and AMH, impaired seminiferous cord structure and reduced production of Sertoli cell factors Inhibin B and AMH.

### Inhibiting WNT/β-catenin signalling in ex vivo cultures of human fetal ovaries had only minor effects on granulosa cell identity and function

**Fig. 4:**
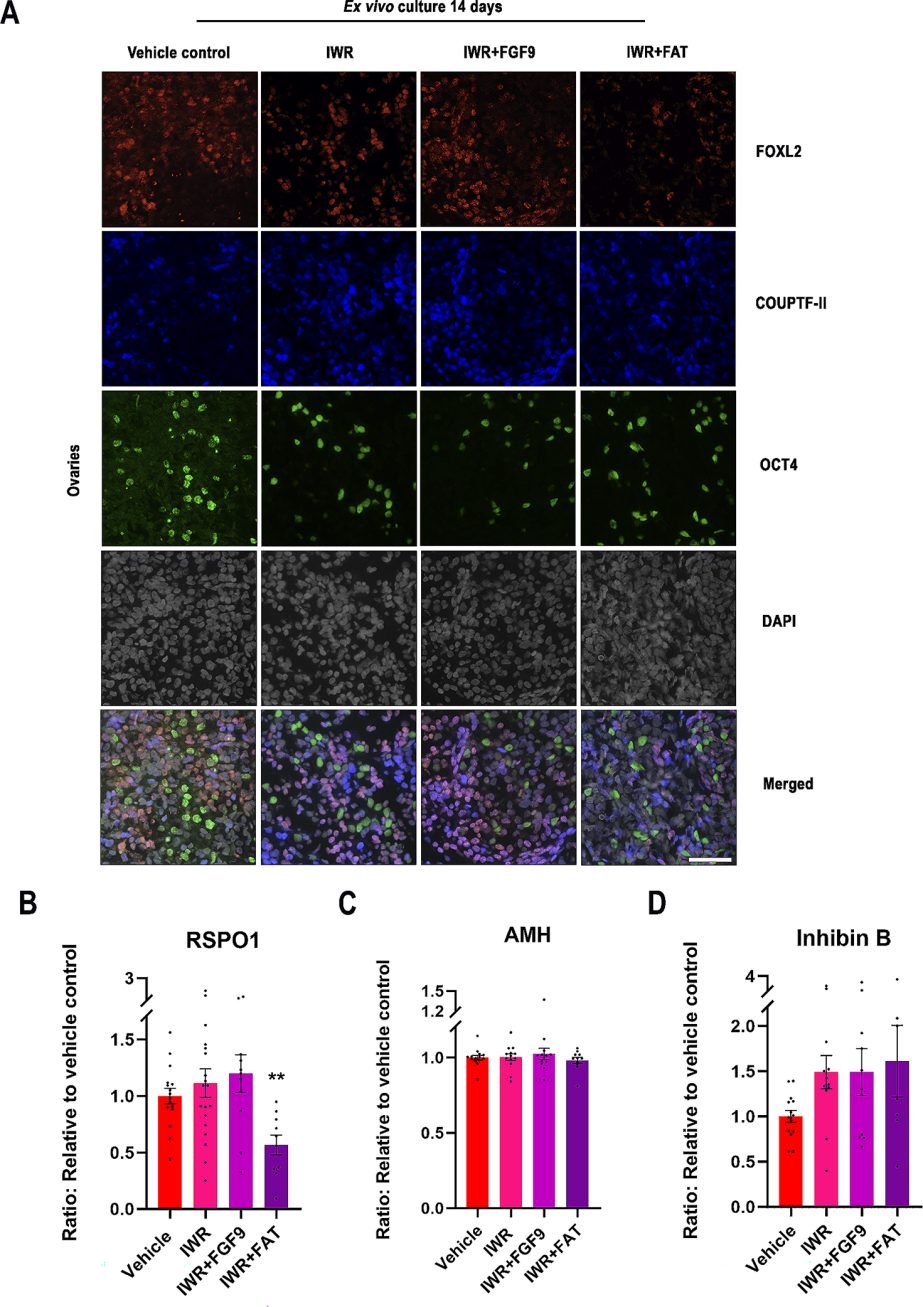
Inhibiting WNT/β-catenin signalling in ex vivo cultures of human fetal ovaries had only minor effects on granulosa cell identity and function. **(A)** Representative images of triple immunofluorescence staining of FOXL2 (Granulosa cell marker, red), (COUPTF-II (Interstitial cell marker, blue), OCT4 (oogonia marker, green) and DAPI (grey) in ex vivo cultured fetal ovaries treated with IWR (1µM), IWR + FGF9: IWR-1 (1µM) + FGF9 (50 ng/ml) or IWR + FAT: IWR-1 (1µM) + FGF9 (50 ng/ml) + Activin A (25 ng/ml) + Activin B (25 ng/ml) + TGFβ (25 ng/ml). Age of fetal samples shown (at start of experiment): Vehicle control 8 + 0 PCW; IWR 7 + 6 PCW; IWR + FGF9 7 + 6 PCW; IWR + FAT 7 + 6 PCW. Scale bar corresponds to 50 μm. **(B)** Secretion of RSPO1 measured in media from ex vivo cultured fetal ovaries treated with IWR (*n* = 19), IWR + FGF9 (*n* = 11) or IWR + FAT (*n* = 10). **(C)** AMH measured in media from ex vivo cultured fetal ovaries treated with IWR (*n* = 13), IWR + FGF9 (*n* = 8) or IWR + FAT (*n* = 7). **(D)** Inhibin B measured in media from ex vivo cultured fetal ovaries treated with IWR (*n* = 13), IWR + FGF9 (*n* = 12) or IWR + FAT (*n* = 11). Results are shown as fold change compared to internal vehicle control with data presented as mean ± SEM with individual datapoints included. Asterisk indicates statistical significance compared to vehicle control with ** *P* < 0.01

Next, the effects on the granulosa cell function and identity were examined after inhibition of WNT/β-catenin signalling in the ex vivo cultured fetal ovaries. Expression of the granulosa cell marker FOXL2 (nuclear), interstitial cell marker COUPTFII (nuclear) and the oogonia marker OCT4 (nuclear) was examined (Fig. 4A). Treatment with IWR and IWR + FGF9 had no apparent effect on FOXL2 expression in the ex vivo cultured ovaries, but FOXL2 expression appeared to be reduced following treatment with IWR + FAT (Fig. 4A). No pronounced alterations in the expression of OCT4 or COUPTFII was observed following any of the treatments (Fig. 4A).

Inhibition of WNT/β-catenin signalling in fetal ovary cultures reduced the secretion of RSPO1, although only after treatment with IWR + FAT (44%, *p* < 0.01) compared with vehicle controls (Fig. 4B), while no effects on RSPO1 secretion were found after treatment with IWR or IWR + FGF9 (Fig. 4B). Treatment with IWR, IWR + FGF9 and IWR + FAT did not affect the production of AMH (Fig. 4C), and Inhibin B (Fig. 4D) compared to vehicle controls, although there was a tendency towards increased Inhibin B secretion following all treatments but this was not statistically significant. Together, these results suggest that inhibition of WNT/β-catenin signalling in fetal ovaries only slightly alters granulosa cell function with reduced secretion of RSPO1 and a tendency towards increased secretion of Inhibin B, although these effects were not consistently found in all treatment groups. Notably, no expression of AMH nor SOX9 was observed in ovaries following any of the treatments, indicating that inhibition of WNT/β-catenin signalling at this developmental time-point was not sufficient to induce granulosa-to-Sertoli cell trans-differentiation (Supplementary Fig. 3).

### Stimulation of WNT/β-catenin signalling in ex vivo cultures of human fetal testes reduce the production of androgens and INSL3

**Fig. 5:**
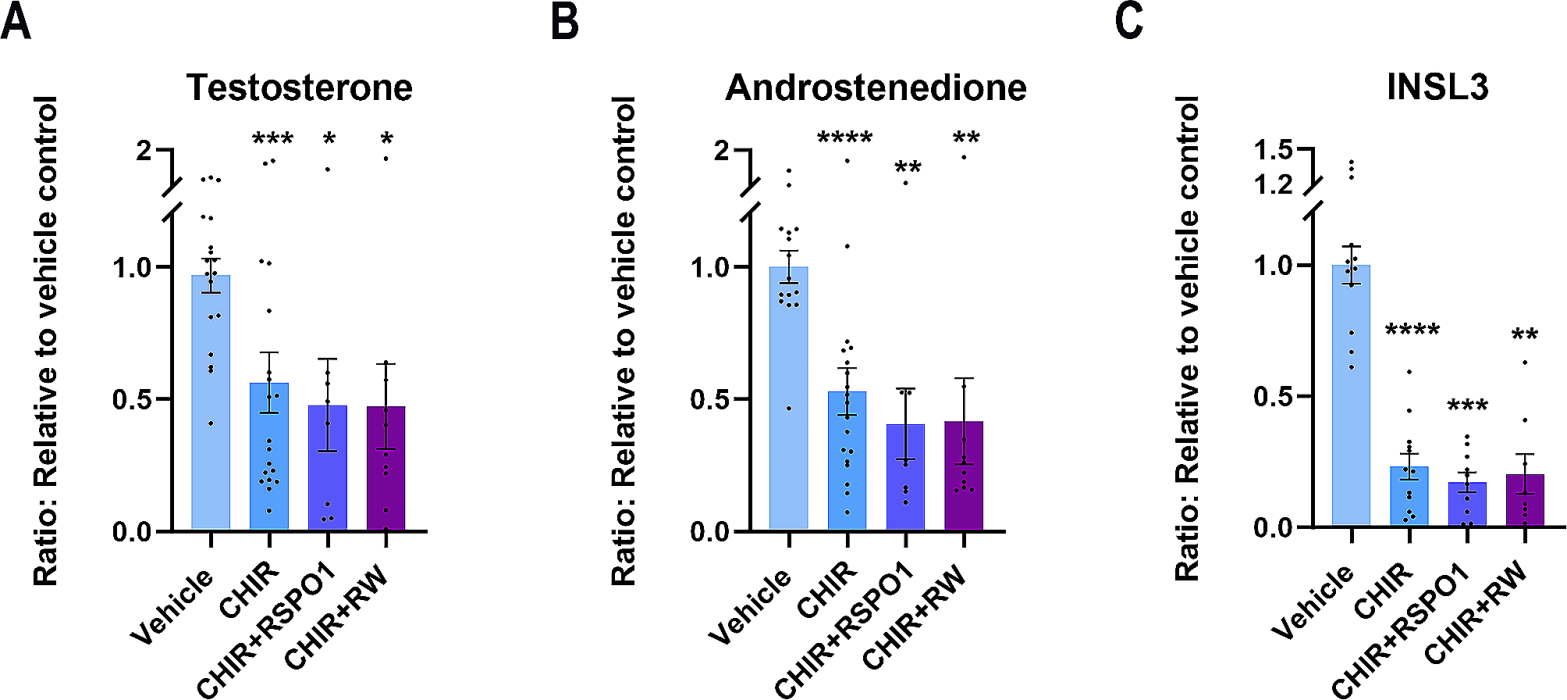
Stimulation of WNT/β-catenin signalling in ex vivo cultures of human fetal testes reduced the production of androgens and INSL3. **(A)** Secretion of testosterone measured in media from ex vivo cultured fetal testes treated with CHIR (*n* = 19), CHIR + RSPO1 (*n* = 8) or CHIR + RW (*n* = 10) **(B)** androstenedione measured in media from ex vivo cultured fetal testes treated with CHIR (*n* = 19), CHIR + RSPO1 (*n* = 8) or CHIR + RW (*n* = 10). **(C)** INSL3 measured in media from ex vivo cultured fetal testes treated with CHIR (*n* = 12), CHIR + RSPO1 (*n* = 10) or CHIR + RW (*n* = 8). Results are shown as fold change compared to internal vehicle control with data presented as mean ± SEM with individual datapoints included. Asterisk indicates statistical significance compared to vehicle control with * *P* < 0.05, ** *P* < 0.01, *** *P* < 0.001 and **** *P* < 0.0001

Subsequently, the effects on Leydig cell function were examined following promotion of WNT/β-catenin signalling in fetal testes cultures. Stimulation of WNT/β-catenin signalling by treatment with CHIR, CHIR + RSPO1 or CHIR + RW significantly reduced the production of testosterone (44%, *p* < 0.001; 53%, *p* < 0.05 and 54%, *p* < 0.05, respectively) (Fig. 5A) and androstenedione (48%, *p* < 0.0001; 60%, *p* < 0.01 and 59%, *p* < 0.01, respectively) (Fig. 5B), while there was no effect on the secretion of DHEAS compared to vehicle controls (Supplementary Fig. 4). Interestingly, the secretion of INSL3 was also severely reduced after treatment with CHIR (77%, *p* < 0.0001), CHIR + RSPO1 (83%, *p* < 0.001) and CHIR + RW in the fetal testis (80%, *p* < 0.01) compared to vehicle controls (Fig. 5C). Together, these results suggest that the promotion of WNT/β-catenin signalling in Sertoli cells in human fetal testes also severely affects Leydig cell function as evident by the reduced production of androgens (testosterone and androstenedione) and INSL3.

### Stimulation of WNT/β-catenin signalling in ex vivo cultures of human fetal testes reduced the sertoli cell number and impaired seminiferous cord structures in 3D imaging analyses

**Fig. 6:**
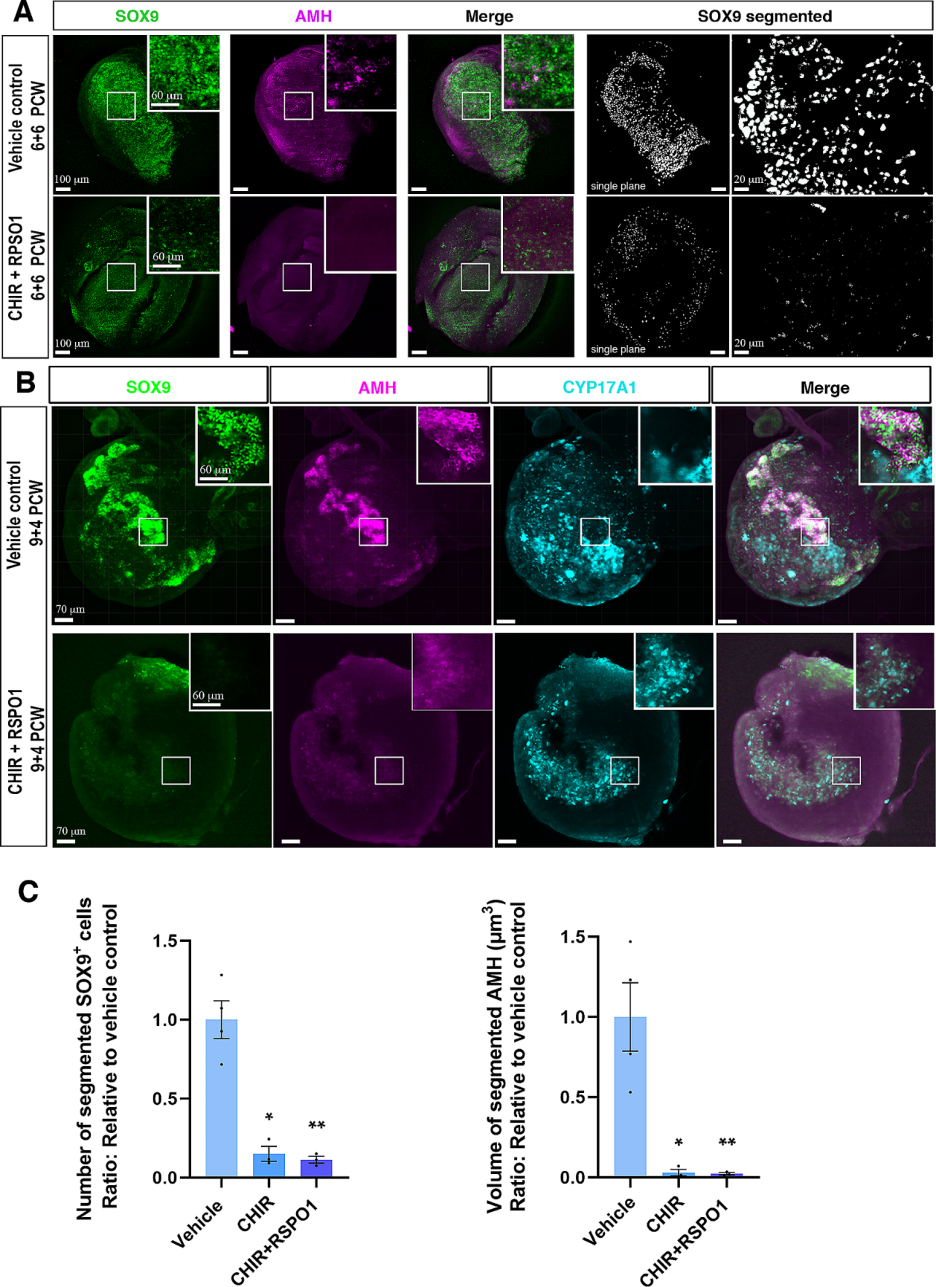
Stimulation of WNT/β-catenin signalling in ex vivo cultures of human fetal testes reduced Sertoli cell number and impaired seminiferous cord structures in 3D imaging analyses. **A**) Representative images of SOX9 and AMH *in toto*-immunostaining of ex vivo cultured fetal testes (6 + 6 PCW) treated or not with CHIR (3 µM) + RSPO1 (100 ng/ml). Right-hand panels are images of single-plane SOX9 segmented areas. **B**) Representative images of SOX9, AMH and CYP17A1 *in toto*-immunostaining of ex vivo cultured fetal testes (9 + 4 PCW) treated or not with CHIR (3 µM) + RSPO1 (100 ng/ml). **C**) Automated quantitative analysis was performed using a machine-learning based software to assess, respectively, the total number of SOX9 nuclei and the volume of AMH-positive segmented areas in control testes, CHIR-treated testes and CHIR + RSPO1-treated testes with *n* = 3 (fetal testes age: 6 + 6, 9 + 4 and 9 + 6 PCW). Results are shown as fold change compared to internal vehicle control with data presented as mean ± SEM with individual datapoints included. Asterisk indicates statistical significance compared to vehicle control with * *P* < 0.05 and ** *P* < 0.01

To further examine the consequences of promoting WNT/β-catenin signalling in human fetal testes, 3D imaging analyses [34] were applied. The development of Sertoli cells (SOX9^+^ and AMH^+^) as well as seminiferous cord structures were visualised in human fetal testes cultures. In vehicle control treated fetal testes aged 6 + 6 PCW, expression of SOX9 and to some extent AMH was found within the seminiferous cords (Fig. 6A, Supplementary Video 1), whereas in testes treated with CHIR + RSPO1 the expression of SOX9 and AMH appeared to be severely reduced (Fig. 6A, Supplementary Video 2). In accordance with the immunofluorescence staining, the 3D imaging analysis demonstrated that treatment with CHIR + RSPO1 resulted in impaired seminiferous cord structure as also evident from the SOX9 nuclei segmentation (Fig. 6A).

In experiments with samples from later developmental stages (9 + 4 PCW and 9 + 6 PCW), it was similarly demonstrated that both the Sertoli cells (SOX9^+^ and AMH^+^) and seminiferous cord structures were altered following treatment with CHIR and CHIR + RSPO1 (Fig. 6B). Inclusion of the fetal Leydig cell marker (CYP17A1^+^) demonstrated their presence in the interstitium in vehicle control treated samples (Fig. 6B). Intriguingly, treatment with CHIR + RSPO1 (Fig. 6B) did not result in altered CYP17A1 expression despite the almost completely abolished expression of SOX9 and AMH in Sertoli cells and the impaired seminiferous cord structures. Quantitative analysis revealed a significant reduction of SOX9^+^ Sertoli cells in the CHIR (85%, *p* < 0.05) and CHIR + RSPO1 (89%, *p* < 0.01) treated samples as compared to vehicle controls (Fig. 6C, Supplementary Video 3 and 4). Similarly, an almost complete loss of AMH expression was found in the CHIR (98%, *p* < 0.05) and CHIR + RSPO1 (98%, *p* < 0.01) treated samples compared to vehicle control testes cultures (Fig. 6C).

### Inhibition of WNT/β-catenin signalling in ex vivo cultures of human fetal ovaries only slightly affect steroid production

**Fig. 7:**
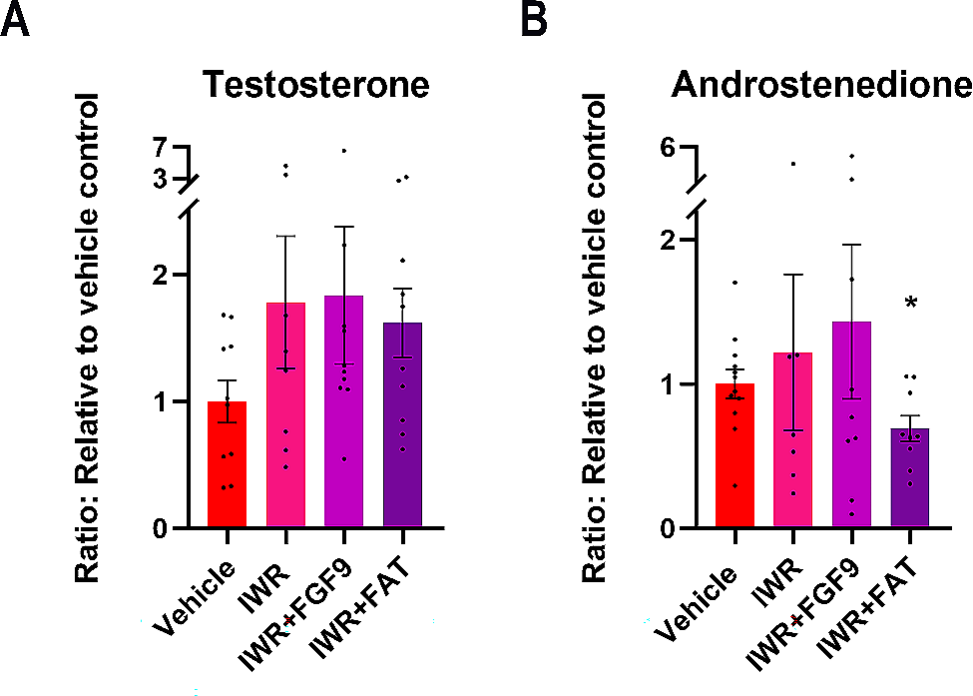
Inhibition of WNT/β-catenin signalling in ex vivo cultures of human fetal ovaries only slightly affect steroid production. **(A)** Secretion of testosterone measured in media from ex vivo cultured fetal ovaries treated with IWR (*n* = 8), IWR + FGF9 (*n* = 10) or IWR + FAT (*n* = 10) and **(B)** androstenedione measured in media from ex vivo cultured fetal ovaries treated with IWR (*n* = 7), IWR + FGF9 (*n* = 9) or IWR + FAT (*n* = 9). Results are shown as fold change compared to internal vehicle control with data presented as mean ± SEM with individual datapoints included. Asterisk indicates statistical significance compared to vehicle control with * *P* < 0.05

Inhibition of WNT/β-catenin signalling in fetal ovary cultures after treatment with IWR, IWR + FGF9 or IWR + FAT appeared to increase the secretion of testosterone compared to vehicle controls, but this effect was not statistically significant (Fig. 7A). Also, there was no effect on androstenedione levels after treatment with IWR and IWR + FGF9 treatment, while a significant reduction (31%, *p* < 0.05) was found after IWR + FAT treatment (Fig. 7B). The production of INSL3 was not detectable in culture media from the majority (12 of 16) of samples analysed from fetal ovaries analysed regardless of treatment. Together, suggesting that the steroidogenic function of the fetal ovaries was only slightly affected after inhibition of WNT/β-catenin signalling.

### Manipulation of WNT/β-catenin signalling in ex vivo cultures of human fetal gonads severely reduce the number of germ cells in testes but not ovaries

**Fig. 8:**
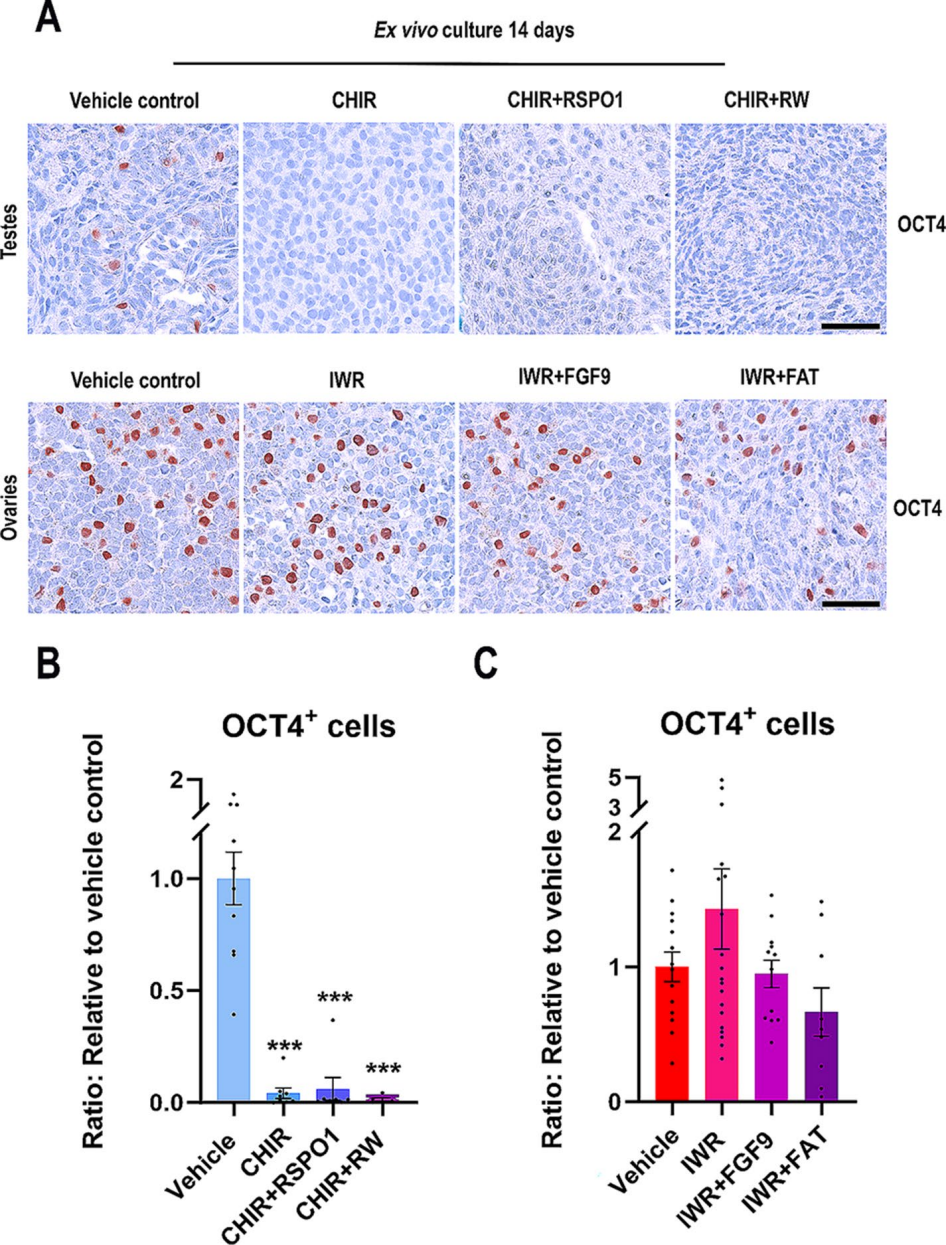
Manipulation of WNT/β-catenin signalling in ex vivo cultures of human fetal gonads severely reduce the number of germ cells in testes but not ovaries. **(A)** Representative images of OCT4 immunostaining in ex vivo cultured fetal testes treated with CHIR (3 µM) or CHIR + RW: CHIR (3 µM) + RSPO1 (100 ng/ml) + WNT4 (100 ng/ml) and ex vivo cultured fetal ovaries treated with IWR (1µM), IWR + FGF9: IWR-1 (1µM) + FGF9 (50 ng/ml) or IWR + FAT: IWR-1 (1µM) + FGF9 (50 ng/ml) + Activin A (25 ng/ml) + Activin B (25 ng/ml) + TGFβ (25 ng/ml). Counterstaining was performed with Mayer’s haematoxylin; scale bar 50 μm. Age of fetal samples shown (at start of experiment): Vehicle control 9 + 0 PCW; CHIR 8 + 2 PCW; CHIR + RSPO1 7 + 2 PCW; CHIR + RW 9 + 0 PCW for testes samples and vehicle control 7 + 0 PCW; IWR 7 + 2 PCW; IWR + FGF9 7 + 1 PCW; IWR + FAT 7 + 6 PCW for ovarian samples. **(B)** Quantification of the number of OCT4^+^ cells/mm^2^ in ex vivo cultured fetal testes treated with CHIR (*n* = 8), CHIR + RSPO1 (*n* = 7) or CHIR + RW (*n* = 5). Results are shown as fold change compared to internal vehicle control with data presented as mean ± SEM with individual datapoints included. Asterisk indicates statistical significance compared to vehicle control with *** *P* < 0.001. **(C)** Quantification of the number of OCT4^+^ cells/mm^2^ in ex vivo cultured fetal ovaries treated with IWR (*n* = 19), IWR + FGF9 (*n* = 12) or IWR + FAT (*n* = 9). Results are shown as fold change compared to internal vehicle control with data presented as mean ± SEM with individual datapoints included

Lastly, the effect on germ cell number (gonocytes and oogonia, both OCT4^+^) was examined after manipulation of WNT/β-catenin signalling. In vehicle control treated fetal testes, OCT4^+^ gonocytes were clearly present within the seminiferous cords, while after stimulation of WNT/β-catenin signalling by CHIR, CHIR + RSPO1 and CHIR + RW the number of OCT4^+^ gonocytes appeared to be reduced (Fig. 8A). Likewise, in vehicle control treated fetal ovaries, numerous OCT4^+^ oogonia were present and this was also observed in ovary cultures following treatment with IWR, IWR + FGF9 and IWR + FAT (Fig. 8A). Quantification of OCT4^+^ gonocytes in the fetal testes demonstrated that the number of OCT4^+^ cells/mm^2^ was indeed reduced after treatment with CHIR, CHIR + RSPO1 and CHIR + RW (all 99.9%, *p* < 0.001) (Fig. 8B). Importantly, no effect on the number of OCT4^+^ cells/mm^2^ was observed in fetal ovaries after treatment with CHIR (Supplementary Fig. 5A), suggesting that loss of gonocytes is a consequence of impaired sex-specific differentiation of the somatic cells following promotion of WNT/β-catenin signalling and not a direct cytotoxic effect of CHIR treatment. No effects on the number of OCT4^+^ cells/mm^2^ was observed after inhibition of WNT/β-catenin signalling in fetal ovary cultures following treatment with IWR, IWR + FGF9 or IWR + FAT when compared to vehicle controls (Fig. 8B). Likewise, no effect on the number of OCT4^+^ cells/mm^2^ was observed for fetal testes cultured with IWR (Supplementary Fig. 5B). Together this suggests that stimulation of WNT/β-catenin signalling in human fetal testis cultures severely reduced the number of gonocytes, although this appeared to be an indirect effect. In contrast, the number of oogonia in fetal ovaries were unaffected by inhibition of WNT/β-catenin signalling.

## Discussion

In recent years, an increased understanding of sex-specific human gonadal development has been achieved although the underlying mechanisms have so far mainly been examined in mouse models. Particularly, the mechanisms directing human ovarian differentiation remain largely unexplored. This study investigated whether manipulation of WNT4/β-catenin signalling in the supporting cell lineages affected sex-specific gonadal development and function in ex vivo cultures of human fetal testes and ovaries. The promotion of WNT4/β-catenin signalling in human fetal testes reduced the expression of Sertoli cell markers (SOX9 and AMH) as well as secretion of Sertoli cell factors AMH and Inhibin B. Additional effects on the somatic niche was evident from the loss of seminiferous cord structures and the subsequent pronounced reduction in the number of OCT4^+^ gonocytes. Importantly, the promotion of WNT4/β-catenin signalling in human fetal testes also severely reduced the production of androgens and INSL3, suggesting that Leydig cell differentiation and/or function have also been impaired. In contrast, the inhibition of WNT4/β-catenin signalling in ex vivo cultured human fetal ovaries did not result in severe effects on ovarian development or function. Although a reduced secretion of RSPO1 and a tendency towards increased production of Inhibin B and testosterone was observed, these were minor alterations in somatic cell function. Together our study demonstrates the involvement of WNT4/β-catenin signalling in the sex-specific differentiation of the somatic cells in human fetal gonads, and in particular the importance of sufficiently inhibiting the WNT4/β-catenin signalling pathway during fetal testis development.

The severely affected Sertoli cell identity found after stimulation of WNT/β-catenin signalling in the fetal testis cultures, including reduced expression of Sertoli cell markers, reduced secretion of Sertoli cell factors and presence of no or few seminiferous structures was similar to effects previously reported after inhibition of Nodal/Activin and Nodal signalling in ex vivo cultured human fetal testes [28]. The observed loss of SOX9 and AMH expression as well as impaired cord structure has also previously been described in XY mouse gonads in which β-catenin expression was stabilised in the supporting cell lineage [14]. Thereby suggesting that repression of β-catenin signalling is important to ensure normal Sertoli cell differentiation in both mouse and human testicular development. The observed lack of seminiferous cord structures following promotion of WNT/β-catenin signalling could in principle be due to either lack of seminiferous cord formation, disintegration of already formed cords or a combination. However, since seminiferous cord formation is progressing during the developmental time-points examined in the present study it is most likely that treatment promoting WNT/β-catenin signalling in the human fetal testes results in the disruption or delay in seminiferous cord formation.

The loss of OCT4-positive gonocytes in fetal testes observed after stimulation of WNT/β-catenin signalling was most likely the result of an altered somatic niche and not a direct effect on the germ cells. Since gonocytes are normally supported by the Sertoli cells within the seminiferous cords, the disruption of seminiferous cord structure appears to have negatively affected germ cell survival. In accordance, germ cell loss was also observed after disruption of seminiferous cords following inhibition of Nodal/Activin and Nodal signalling in ex vivo cultured fetal testes [28]. Despite the pronounced germ cell loss, no increase in apoptosis was observed following stimulation of WNT/β-catenin signalling in human fetal testes cultures. Although an overall reduction in proliferation was found after promotion of WNT/β-catenin signalling it is likely that the majority of gonocytes were lost via apoptosis prior to the examined time-point since this was observed in our previous study following inhibition of Nodal/Activin signalling in human fetal testes using a similar experimental approach [28]. Here, apoptosis was examined after both 7- and 14-days of culture with a higher number of apoptotic cells observed after 7-days of culture indicating a rapid loss of germ cells [28]. Interestingly, the stimulation of WNT4/β-catenin signalling in human fetal testes also severely affected the Leydig cell function resulting in a reduced production of testosterone, androstenedione, and INSL3. These results are in agreement with previous studies in mice demonstrating that WNT4 act as a repressor of steroidogenesis during development of the ovaries [20, 40]. The observation that the development and function of the steroidogenic cell lineage appeared to be more severely affected by the stimulation of WNT4/β-catenin signalling than the supporting cells could be due to the difference in the timing of cell lineage differentiation. Fetal Leydig cell differentiation relies on signalling from the supporting cells and in particular desert hedgehog (DHH) signalling has been shown to be important in mice [41–43]. Therefore, loss of Sertoli cell identity following stimulation of WNT/β-catenin signalling may result in reduced DHH signalling, which may consequently lead to less effective promotion of fetal Leydig cell differentiation. However, the precise role of DHH signalling in the promotion of Leydig cell differentiation remains to be elucidated in human fetal testes. Also, due to the timing of WNT/β-catenin manipulation in the ex vivo cultured gonads, with the youngest samples aged 6 + 2 PCW, it is reasonable to assume that the supporting cell lineage differentiation had already been initiated prior to the initiation of treatments. Notably, despite the observed reduction in testosterone and androstenedione production no difference in DHEAS levels were observed in the fetal testis cultures after stimulation of WNT/β-catenin, which could suggest effects on HSD3B and HSD17B activity rather than CYP11A1 and CYP17A1. This notion is supported by 3D imaging analysis showing no apparent difference in expression level or pattern of CYP17A1 following stimulation of WNT/β-catenin signalling, although this notion should be cautiously interpreted since the number and differentiation status of the Leydig cells were not examined. Interestingly, it is the Sertoli cells (not fetal Leydig cells) that express HSD17B during fetal development and thus mediates the last step of testosterone biosynthesis [44–47], which is consistent with the observed steroidogenic profile and the effects on both cell types following promotion of WNT/β-catenin signalling. The impaired Leydig cell function in ex vivo cultured fetal testis after stimulation of WNT/β-catenin signalling was also evident from the severely reduced secretion of INSL3. Despite INSL3 being well-known to promote testicular descent [48] its function within the developing testes is not understood in detail, but the observed reduction in the production of INSL3 and androgens following stimulation of WNT/β-catenin signalling in fetal testes highlights the importance of repressing this signalling pathway during normal sex-specific differentiation of human fetal testes. Although it was not possible in the present study to distinguish whether the reduced androgen and INSL3 production was due to a partial loss of the Leydig cell population, delayed Leydig cell differentiation, reduced steroidogenic activity or a combination.

In fetal ovaries, inhibition of WNT/β-catenin signalling affected granulosa cell function only slightly as evident by reduced secretion of RSPO1 after treatment with IWR + FAT and a tendency towards an increased secretion of Inhibin B. This is in contrast to previous results from mouse E11.5 XX gonad cultures, where FAT treatment alone (with doses similar to those used in the present study) was sufficient to promote testicular characteristics including expression of Sertoli cell factors, morphological reorganisation, and mitotic arrest of germ cells [32]. Importantly, FAT-mediated effects were diminished when mouse E12.5 XX gonads were examined [32]. Since the majority of human fetal samples used for ex vivo culture experiments in this study (PCW 6–10) were comparatively slightly older than mouse E11.5 this may at least in part explain the observed minor effects of IWR + FGF9 and IWR + FAT treatments in this study. Importantly, no pronounced alterations in FOXL2 expression were observed together suggesting only minor effects of inhibiting WNT/β-catenin signalling on the granulosa cells. Of notice, a previous study also reported unaltered *Foxl2* expression between wildtype and *Rspo1* knockout mice, thereby suggesting that FOXL2 and WNT/β-catenin may have separate roles during ovarian development [16]. Accordingly, loss of either *RSPO1* or *WNT4* results in 46,XX DSD with complete female-to-male sex reversal or virilisation and Müllerian duct regression, respectively [24, 25, 49, 50]. In contrast, loss of *FOXL2* in 46,XX individuals result in primary ovarian insufficiency [51] but not alterations in ovarian development. In further support of this notion, recent human scRNAseq data demonstrates the existence of two granulosa cell populations during early human fetal ovary development, one FOXL2-positive and one FOXL2-negative [52]. This could suggest that the inhibition of WNT/β-catenin signalling primarily affects the FOXL2-negative population and thus may explain the relatively unaltered FOXL2 expression. Importantly, FOXL2 was the only granulosa cell marker examined in the present study and thus it is not possible to exclude that additional effects were induced but was not detected. In both mice and humans, loss-of-function mutations in *RSPO1* and *WNT4* results in either sex-reversal or virilisation that has been attributed to the loss of repression on steroidogenic enzymes which results in increased androgen production and subsequently androgen actions during fetal development [26, 53, 54]. In accordance, this study found a tendency towards an increase in testosterone secretion after inhibition of WNT/β-catenin signalling, while a significant reduction in testosterone production was observed in fetal testis cultures after promotion of WNT/β-catenin signalling. Furthermore, the tendency towards increased levels of testosterone and reduced levels of androstenedione in fetal ovaries treated with IWR + FAT could indicate a treatment-mediated increase in HSD17B activity although this remains speculative. Of note, the levels of testosterone produced by the fetal ovaries in culture are much lower (~ 2000-fold) compared with the levels of testosterone produced by age-matched cultured fetal testis. The inhibition of WNT/β-catenin signalling in human fetal ovaries may thus result in loss of repression of steroidogenic enzymes as a consequence of an altered somatic niche, which could indicate that somatic cells in fetal ovaries are directed slightly towards a more male-like phenotype. The inhibition of WNT/β-catenin signalling in human fetal ovary cultures also reduced overall cell proliferation, but this did not result in a reduced number of oogonia compared to controls. Thus, underlining that the limited treatment-induced alterations in the somatic niche (supporting and steroidogenic cells) did not extend to the germ cell population.

In conclusion, this study demonstrates that tipping the balance between pro-testis and pro-ovarian signals via manipulation of the WNT/β-catenin signalling pathway disrupts normal sex-specific gonadal development in human fetal samples cultured ex vivo. This was mainly evident in fetal testes where promotion of WNT/β-catenin signalling resulted in impaired Sertoli cell and Leydig cell function as well as loss of seminiferous cord structure. Additionally, these alterations to the somatic niche resulted in a severely reduced number of germ cells. In contrast, the effects on fetal ovary development appeared less pronounced with only minor effects detected. Thus, the present study provides detailed cell type specific information about the consequences of dysregulated WNT/β-catenin signalling on the sex-specific gonadal development in humans. Additionally, this study highlights the importance of sufficient inhibition of the female signalling pathway to ensure proper differentiation and function of the somatic cell lineages during human fetal testis development.

### Electronic supplementary material

Below is the link to the electronic supplementary material.

Supplementary Material 1: Supplementary Figure 1

Supplementary Material 2: Supplementary Figure 2

Supplementary Material 3: Supplementary Figure 3

Supplementary Material 4: Supplementary Figure 4

Supplementary Material 5: Supplementary Figure 5

Supplementary Material 6: Supplemntary Table 1

Supplementary Material 7: Supplementary Table 2

Supplementary Material 8: Supplemntary Table 3

Supplementary Material 9: Supplementary Video 1

Supplementary Material 10: Supplementary Video 2

Supplementary Material 11: Supplementary Video 3

Supplementary Material 12: Supplementary Video 4

Supplementary Material 13

## Data Availability

No datasets were generated or analysed during the current study.

## Abbreviations

CHIR: CHIR99021
DCM: Dichloromethane
DHEAS: Dehydroepiandrosterone sulphate
DHH: Desert hedgehog
DSD: Differences (disorders) of sex development
FAT: FGF9 + Activin A + Activin B + TGFβ
ITS: Insulin-Transferrin-Selenium
IWR: IWR-1
NCS: Normal chicken serum
PBS: Phosphate buffered saline
PBSGT: Permeabilized blocking solution
PCW: Post-conceptional week
PGC: Primordial germ cells
RSD: Relative standard deviation
RT: Room temperature
RW: RSPO1 + WNT4
TBS: Tris buffered saline
