# Supplementary figures and images for: Inhibition of WNT/β-catenin signalling during sex-specific gonadal differentiation is essential for normal human fetal testis development
Source: Cell Commun Signal. 2024 Jun 15;22:330. doi: 10.1186/s12964-024-01704-9 (PMC11180390; doi:10.1186/s12964-024-01704-9)

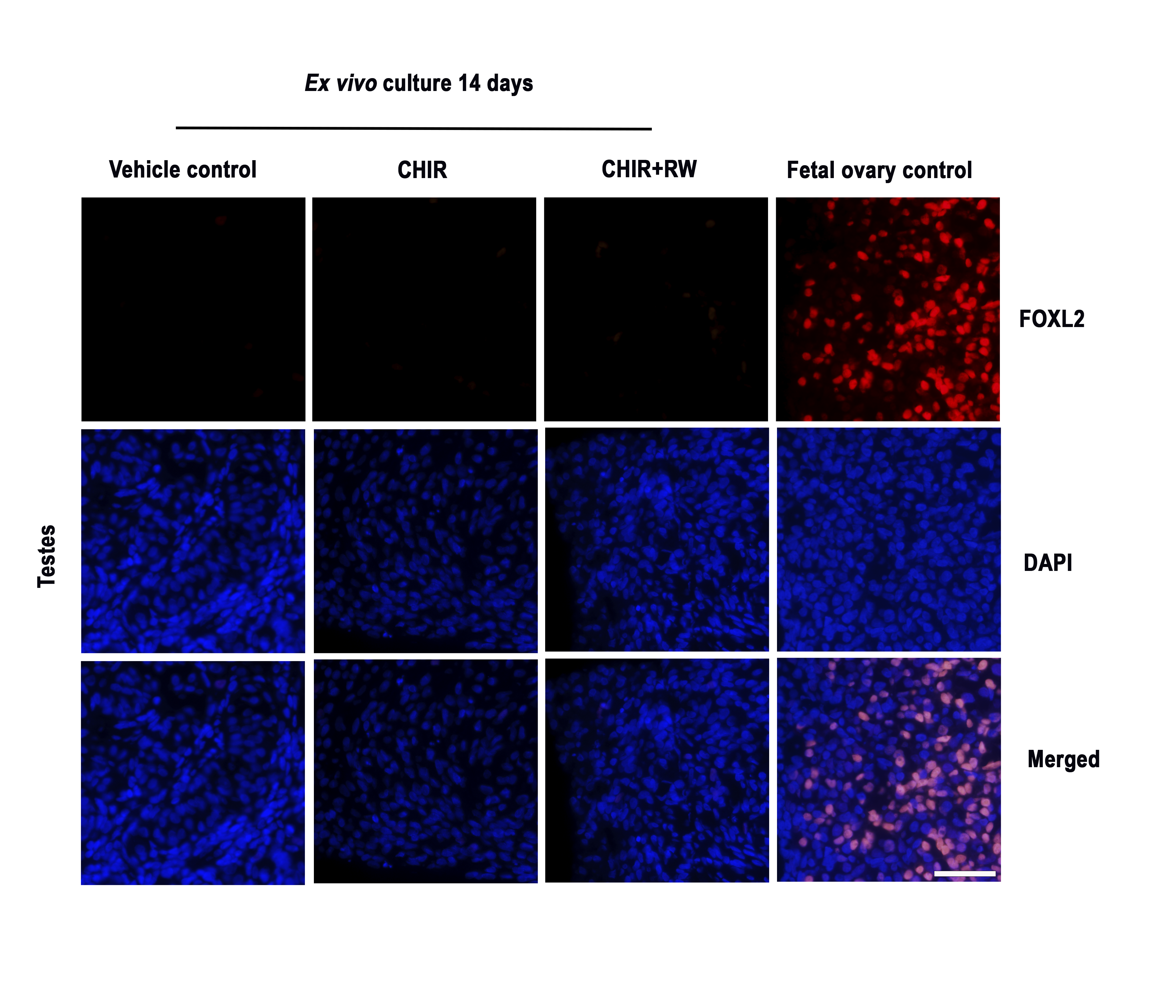

Supplement: Supplementary file 1 — Supplementary Material 1: Supplementary Figure 1 [file 12964_2024_1704_MOESM1_ESM.tif]

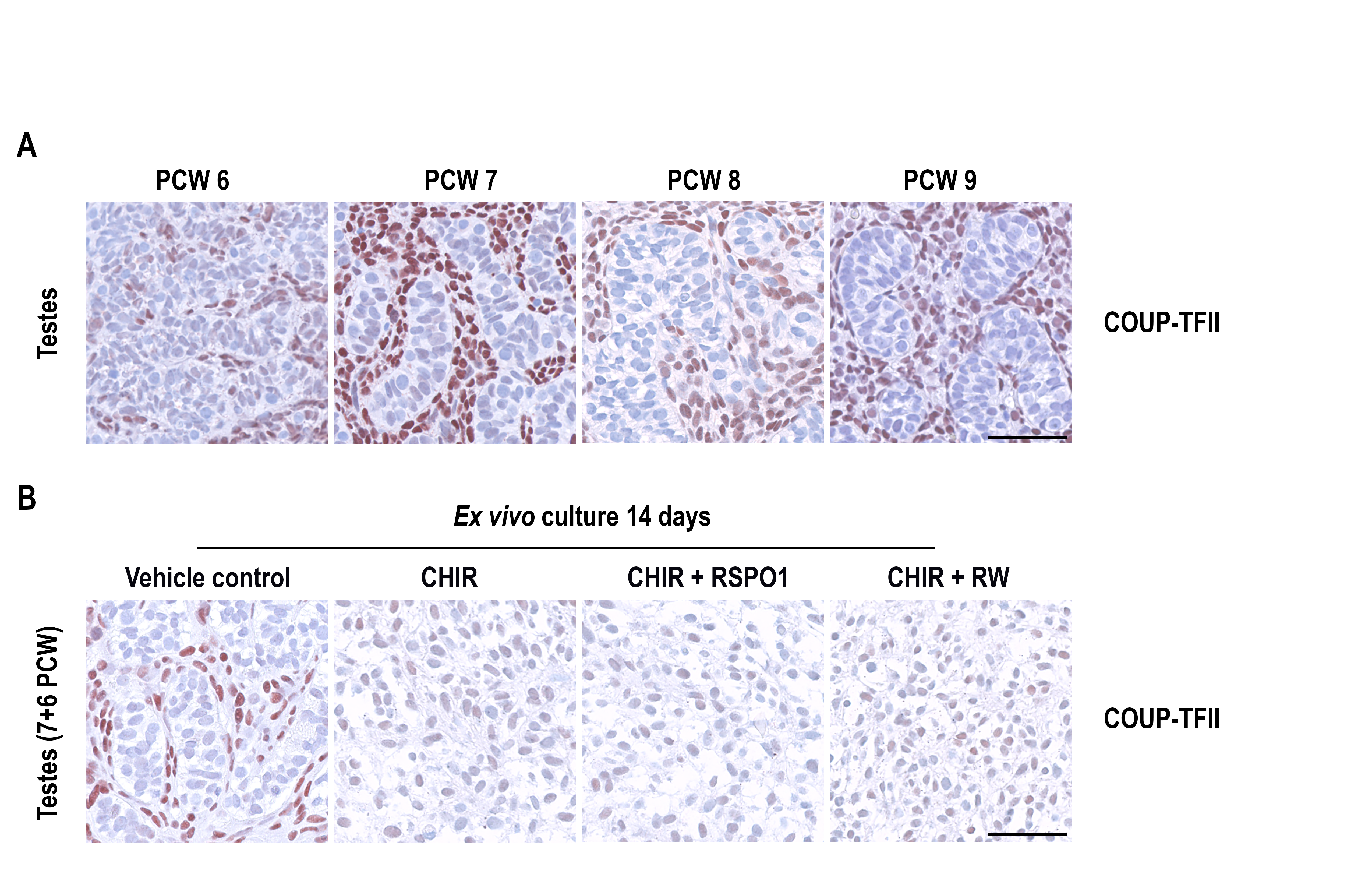

Supplement: Supplementary file 2 — Supplementary Material 2: Supplementary Figure 2 [file 12964_2024_1704_MOESM2_ESM.tif]

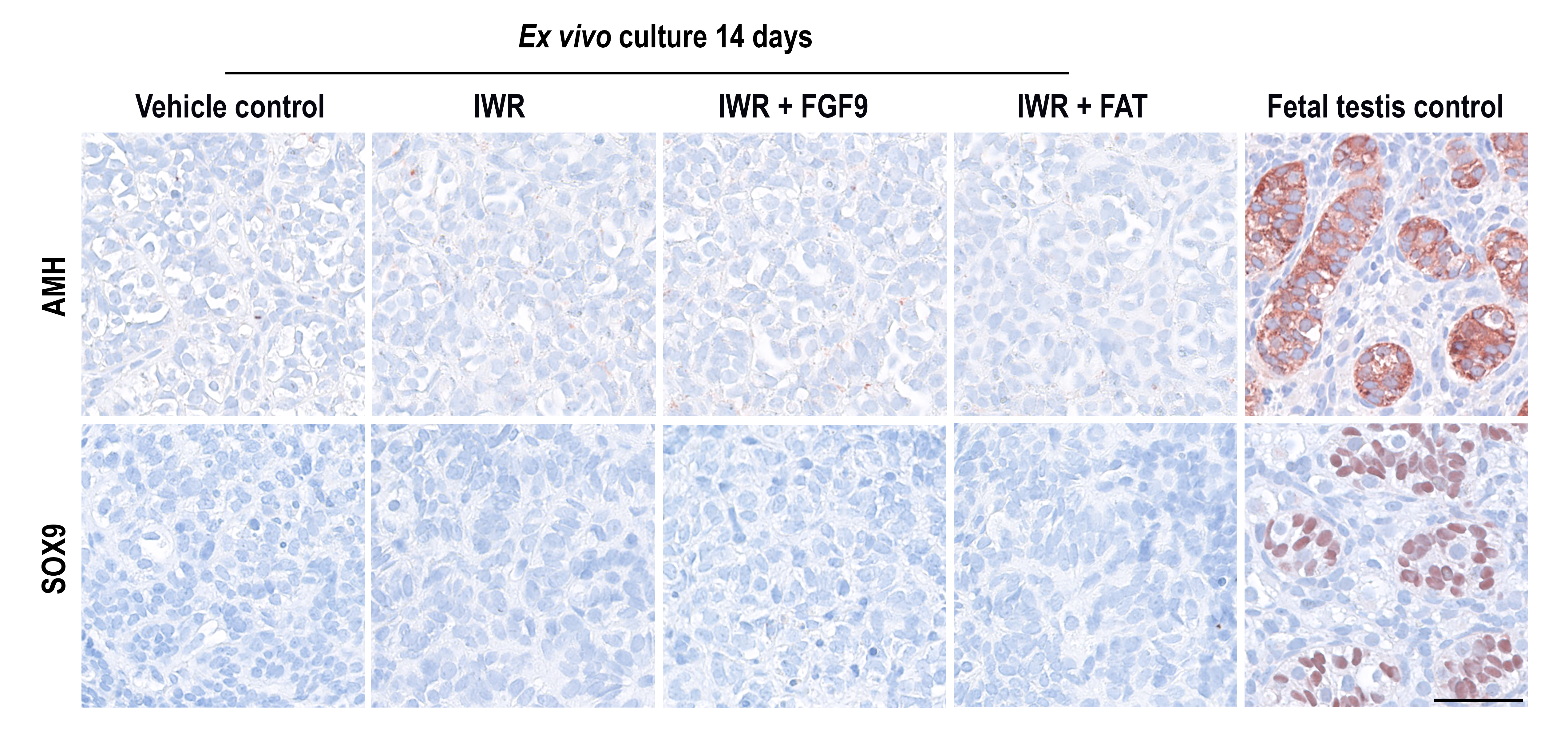

Supplement: Supplementary file 3 — Supplementary Material 3: Supplementary Figure 3 [file 12964_2024_1704_MOESM3_ESM.tif]

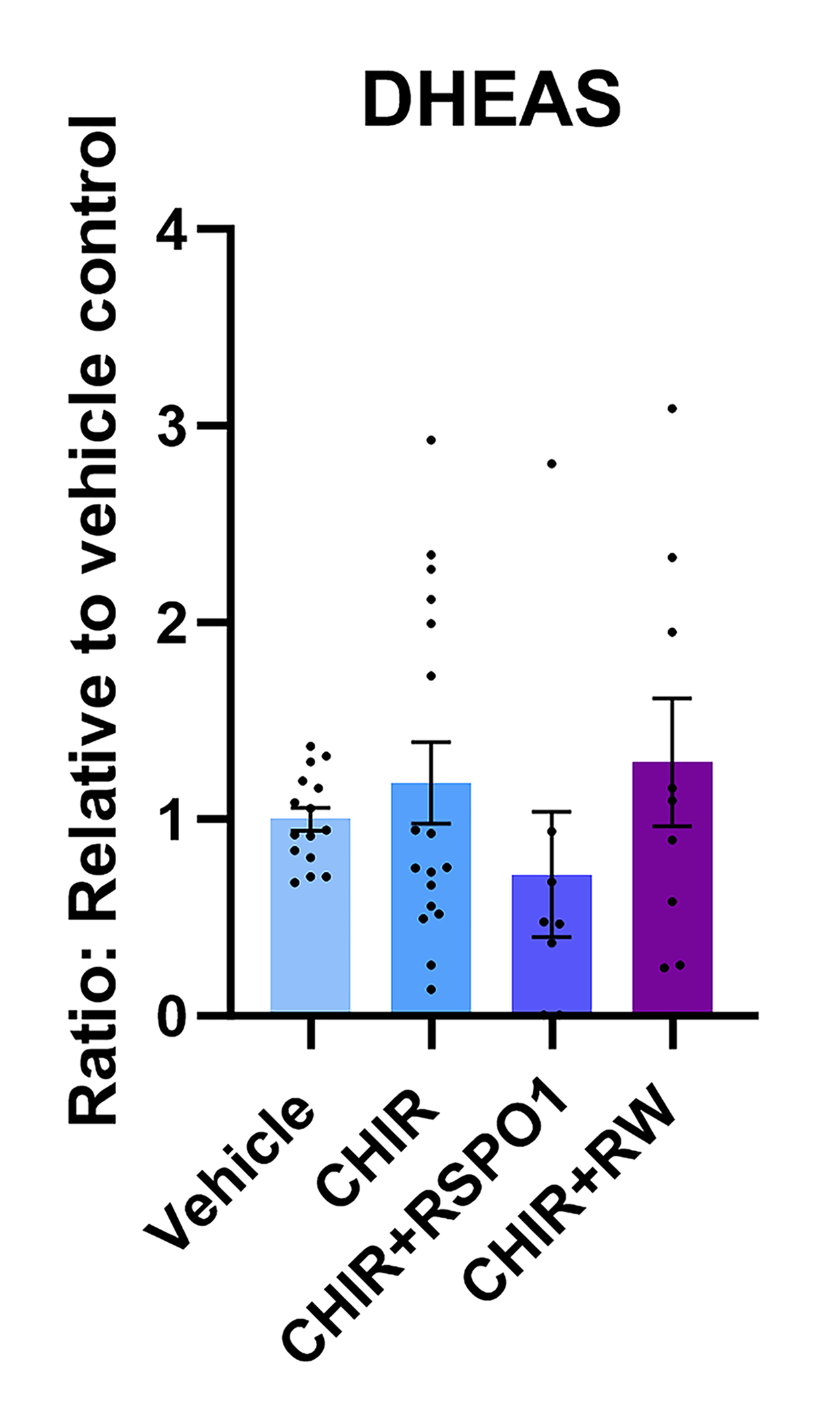

Supplement: Supplementary file 4 — Supplementary Material 4: Supplementary Figure 4 [file 12964_2024_1704_MOESM4_ESM.tif]

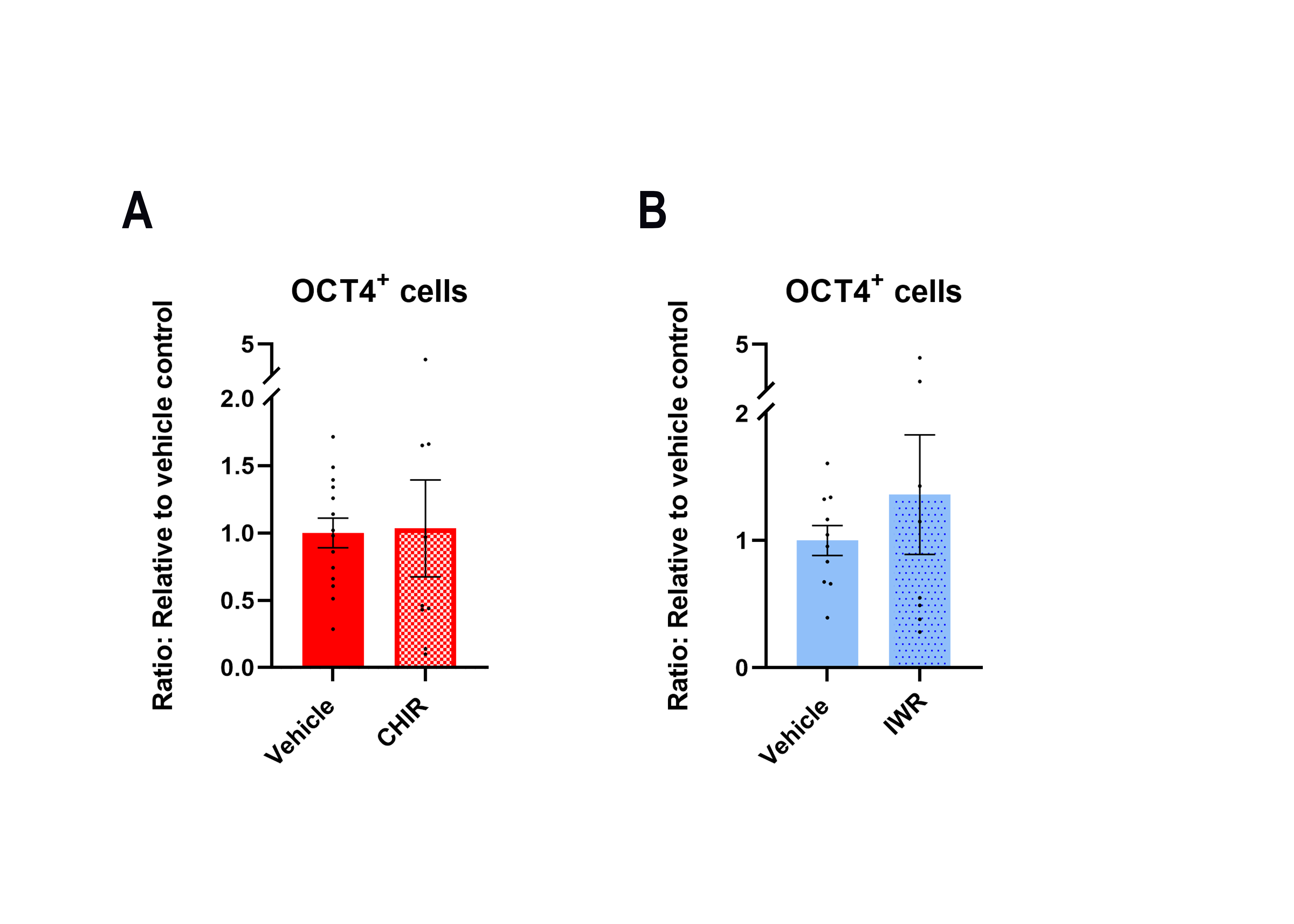

Supplement: Supplementary file 5 — Supplementary Material 5: Supplementary Figure 5 [file 12964_2024_1704_MOESM5_ESM.tif]
