# Supplementary material for: Inhibition of WNT/β-catenin signalling during sex-specific gonadal differentiation is essential for normal human fetal testis development
Source: Cell Commun Signal. 2024 Jun 15;22:330. doi: 10.1186/s12964-024-01704-9 (PMC11180390; doi:10.1186/s12964-024-01704-9)
Supplement: Supplementary file 6 — Supplementary Material 6: Supplemntary Table 1 [file 12964_2024_1704_MOESM6_ESM.docx]

**Supplementary Table 1. Antibodies for immunohistochemistry.**

| **Antibody** | **Dilution** | **Retrieval buffer** | **Company** | **Cat. Number** | **Antigen retrieval** | **RRID** |
| --- | --- | --- | --- | --- | --- | --- |
| OCT4 | 1:250 | TEG | Santa Cruz | Sc-5279 | Microwaving | AB_628051 |
| MAGE-A4 | 1:250 | TEG | Non-commercial | Gift from Prof. Spagnoli | Microwaving | NA |
| AMH | 1:250 | CIT | Santa Cruz | Sc-6886 | Microwaving | AB_649207 |
| SOX9 | 1:800 | CIT | Millipore | AB5535 | Microwaving | AB_2239761 |
| COUP-TFII | 1:200 | CIT | Perseus Proteomics | PP-H7147-00 | Microwaving | AB_2314222 |
| BrdU | 1:100 | CIT | Dako | M0744 | Microwaving | AB_10013600 |
| cPARP | 1:500 | CIT | Cell Signalling | 5625 | Pressure cooker | AB_10699459 |

Antigen retrieval was conducted by microwaving or placing sections in a pressure cooker in the indicated retrieval buffer. TEG buffer: 10 mM Tris, 0.5 mM EGTA, pH 9.0; Citrate (CIT) buffer: 10 mM, pH 6.0. NA: not available.
