# Supplementary material for: Inhibition of WNT/β-catenin signalling during sex-specific gonadal differentiation is essential for normal human fetal testis development
Source: Cell Commun Signal. 2024 Jun 15;22:330. doi: 10.1186/s12964-024-01704-9 (PMC11180390; doi:10.1186/s12964-024-01704-9)
Supplement: Supplementary file 7 — Supplementary Material 7: Supplementary Table 2 [file 12964_2024_1704_MOESM7_ESM.docx]

**Supplementary Table 2. Antibodies for immunofluorescence.**

| **Antibody** | **Dilution** | **Company** | **Cat. Number** | **RRID** |
| --- | --- | --- | --- | --- |
| OCT4 | 1:100 | Santa Cruz | Sc-5279 | AB_628051 |
| SOX9 | 1:1500 | Millipore | AB5535 | AB_2239761 |
| AMH | 1:2000 | Santa Cruz | Sc-6886 | AB_649207 |
| FOXL2 | 1:100 | Non-commercial | Gift from Dr. Wilhelm | AB_2687958 |
| COUPTFII | 1:100 | Perseus Proteomics | PP-H7147-00 | AB_2314222 |
