# Supplementary material for: Inhibition of WNT/β-catenin signalling during sex-specific gonadal differentiation is essential for normal human fetal testis development
Source: Cell Commun Signal. 2024 Jun 15;22:330. doi: 10.1186/s12964-024-01704-9 (PMC11180390; doi:10.1186/s12964-024-01704-9)
Supplement: Supplementary file 8 — Supplementary Material 8: Supplemntary Table 3 [file 12964_2024_1704_MOESM8_ESM.docx]

**Supplementary Table 3. Antibodies for whole-mount immunostaining.**

| **Antibody** | **Dilution** | **Company** | **Cat. Number** | **RRID** |
| --- | --- | --- | --- | --- |
| SOX9 | 1:1000 | Millipore | AB5535 | AB_2239761 |
| AMH | 1:400 | Abcam | Ab24542 | AB_2801539 |
| CYP17A1 | 1:200 | Abcam | Ab48019 | AB_869326 |
