# Supplementary material for: Inhibition of WNT/β-catenin signalling during sex-specific gonadal differentiation is essential for normal human fetal testis development
Source: Cell Commun Signal. 2024 Jun 15;22:330. doi: 10.1186/s12964-024-01704-9 (PMC11180390; doi:10.1186/s12964-024-01704-9)
Supplement: Supplementary file 13 — Supplementary Material 13 [file 12964_2024_1704_MOESM13_ESM.docx]

**Supplemental information titles and legends**

**Supplementary Fig. 1. Effects of promoting WNT/β-catenin signalling in *ex vivo* cultured human fetal testes on FOXL2 expression.** Representative images of immunofluorescence staining of FOXL2 (Granulosa cell marker, red) and DAPI (blue) in an *ex vivo* cultured control fetal ovary and *ex vivo* cultured fetal testes treated with CHIR (3 μM) or CHIR + RW: CHIR (3 μM) + RSPO1 (100 ng/ml) + WNT4 (100 ng/ml). Age of fetal samples shown (at start of experiment): Vehicle control ovary 11 + 2 PCW, Vehicle control testis 11 + 0 PCW; CHIR 11 + 0 PCW; CHIR + RW 11 + 0 PCW. Scale bar corresponds to 50 µm.

**Supplementary Fig. 2. Expression of AMH and SOX9 effects of promoting WNT/β-catenin signalling in *ex vivo* cultured samples. A)** Representative images of immunostaining with the interstitial cell marker COUP-TFII in human fetal testes samples (not cultured) aged 6–9 PCW **B)** Representative images of COUP-TFII immunostaining in *ex vivo* cultured fetal testes treated with CHIR (3 μM) or CHIR + RW: CHIR (3 μM) + RSPO1 (100 ng/ml) + WNT4 (100 ng/ml). Age of fetal samples shown (at start of experiment): Vehicle control 9 + 0 PCW; CHIR 9 + 0 PCW; CHIR + RSPO1 9 + 4 PCW; CHIR + RW 9 + 0 PCW. Counterstaining was performed with Mayer’s haematoxylin; scale bar 50 µm.

**Supplementary Fig. 3. Effects of inhibiting WNT/β-catenin signalling in *ex vivo* cultured human fetal ovaries on AMH and SOX9 expression.** Representative images of immunostaining with the Sertoli cell markers AMH (cytoplasmic) and SOX9 (nuclear) in *ex vivo* cultured fetal ovaries treated with IWR (1 μM), IWR + FGF9: IWR-1 (1 μM) + FGF9 (50 ng/ml) or IWR + FAT: IWR-1 (1 μM) + FGF9 (50 ng/ml) + Activin A (25 ng/ml) + Activin B (25 ng/ml) + TGFβ (25 ng/ml). Age of fetal samples shown (at start of experiment): Vehicle control 8 + 2 PCW; IWR 9 + 3 PCW; IWR + FGF9 8 + 2 PCW; IWR + FAT 8 + 2 PCW. Fetal testis (not cultured, age 8 + 1 PCW) was included as positive control. Counterstaining was performed with Mayer’s haematoxylin; scale bar 50 µm.

**Supplementary Fig. 4. Stimulation of WNT/β-catenin signalling** **in *ex vivo* cultures of human fetal testes did not alter the production of DHEAS.** Secretion of DHEAS measured in media from *ex vivo* cultured fetal testes treated with CHIR (*n = 17*), CHIR + RSPO1 (*n = 8*) or CHIR + RW (*n = 9*). Results are shown as fold change compared to internal vehicle control with data presented as mean ± SEM with individual datapoints included.

**Supplementary Fig. 5. Effects of manipulating WNT/β-catenin signalling on germ cell numbers in** ***ex vivo* cultured human fetal testes and ovaries. A)** Quantification of the number of OCT4^+^ cells/mm^2^ in *ex vivo* cultured fetal ovaries treated with CHIR (3 μM) (*n = 9*). **B)** Quantification of the number of OCT4^+^ cells/mm^2^ in *ex vivo* cultured fetal testes treated with IWR (1 μM) (*n = 8*). Results are shown as fold change compared to internal vehicle control with data presented as mean ± SEM with individual datapoints included.

**Supplementary Video 1. Whole-mount immunostaining and 3D-imaging of *ex vivo* cultured human fetal testes.** Representative video of SOX9 (green) and AMH (magenta) *in toto*-immunostaining of *ex vivo* cultured fetal testes (6 + 6 PCW) vehicle-treated.

**Supplementary Video 2. Whole-mount immunostaining and 3D-imaging of *ex vivo* cultured human fetal testes.** Representative video of SOX9 (green) and AMH (magenta) *in toto*-immunostaining of *ex vivo* cultured fetal testes (6 + 6 PCW) treated with CHIR (3 μM) + RSPO1 (100 ng/ml).

**Supplementary Video 3. SOX9^+^ nuclei segmentation of whole-mount immunostaining and 3D-imaged vehicle-treated *ex vivo* cultured human fetal testes.** Representative video of machine-learning based segmentation of SOX9^+^ nuclei (white) of the *in toto*-immunostaining of *ex vivo* cultured fetal testes (6 + 6 PCW) vehicle-treated.

**Supplementary Video 4. SOX9^+^ nuclei segmentation of whole-mount immunostaining and 3D-imaged CHIR + RSPO1-treated *ex vivo* cultured human fetal testes.** Representative video of machine-learning based segmentation of SOX9^+^ nuclei (white) of the *in toto*-immunostaining of *ex vivo* cultured fetal testes (6 + 6 PCW) treated with CHIR (3 μM) + RSPO1 (100 ng/ml).
